# Supplementary material for: Flexible tungsten disulfide superstructure engineering for efficient alkaline hydrogen evolution in anion exchange membrane water electrolysers
Source: Nat Commun. 2024 Jul 8;15:5702. doi: 10.1038/s41467-024-50117-2 (PMC11231348; doi:10.1038/s41467-024-50117-2)
Supplement: Supplementary file 1 — Supplementary Information [file 41467_2024_50117_MOESM1_ESM.pdf]

Supplementary Information for

Flexible tungsten disulfide superstructure engineering for efficient alkaline hydrogen evolution in anion exchange membrane water electrolysers

Lingbin Xie,[1,2] Longlu Wang,[1]* Xia Liu,[3] Jianmei Chen,[1] Xixing Wen,[1] Weiwei Zhao,[2] Shujuan Liu,[2]* and Qiang Zhao[1,2]*

[1] College of Electronic and Optical Engineering & College of Flexible Electronics (Future Technology), State Key Laboratory of Organic Electronics and Information Displays & Jiangsu Key Laboratory for Biosensors, Nanjing University of Posts & Telecommunications, 9 Wenyuan Road, Nanjing 210023, PR China

[2] Institute of Advanced Materials (IAM) & Institute of Flexible Electronics (Future Technology), Nanjing University of Posts & Telecommunications, 9 Wenyuan Road, Nanjing 210023, PR China

[3] College of Chemistry and Chemical Engineering, Qingdao University, Qingdao 266071, Shandong Province, PR China

E-mail addresses: wanglonglu@njupt.edu.cn (L. Wang), iamsjliu@njupt.edu.cn (S. Liu), iamqzhao@njupt.edu.cn (Q. Zhao)

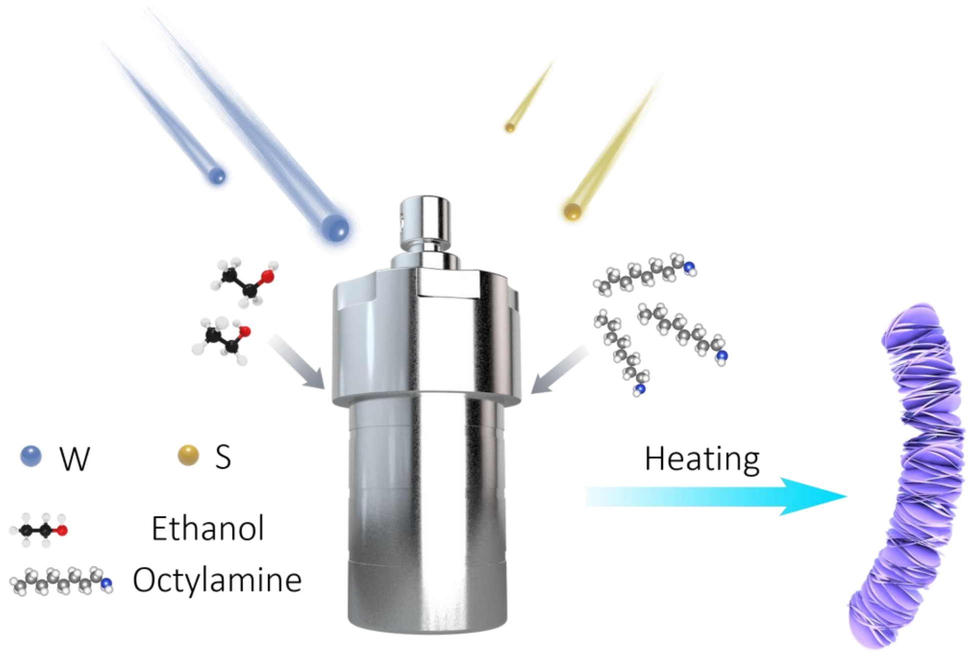

Supplementary Figure 1 | Schematic illustration of the synthesis process.

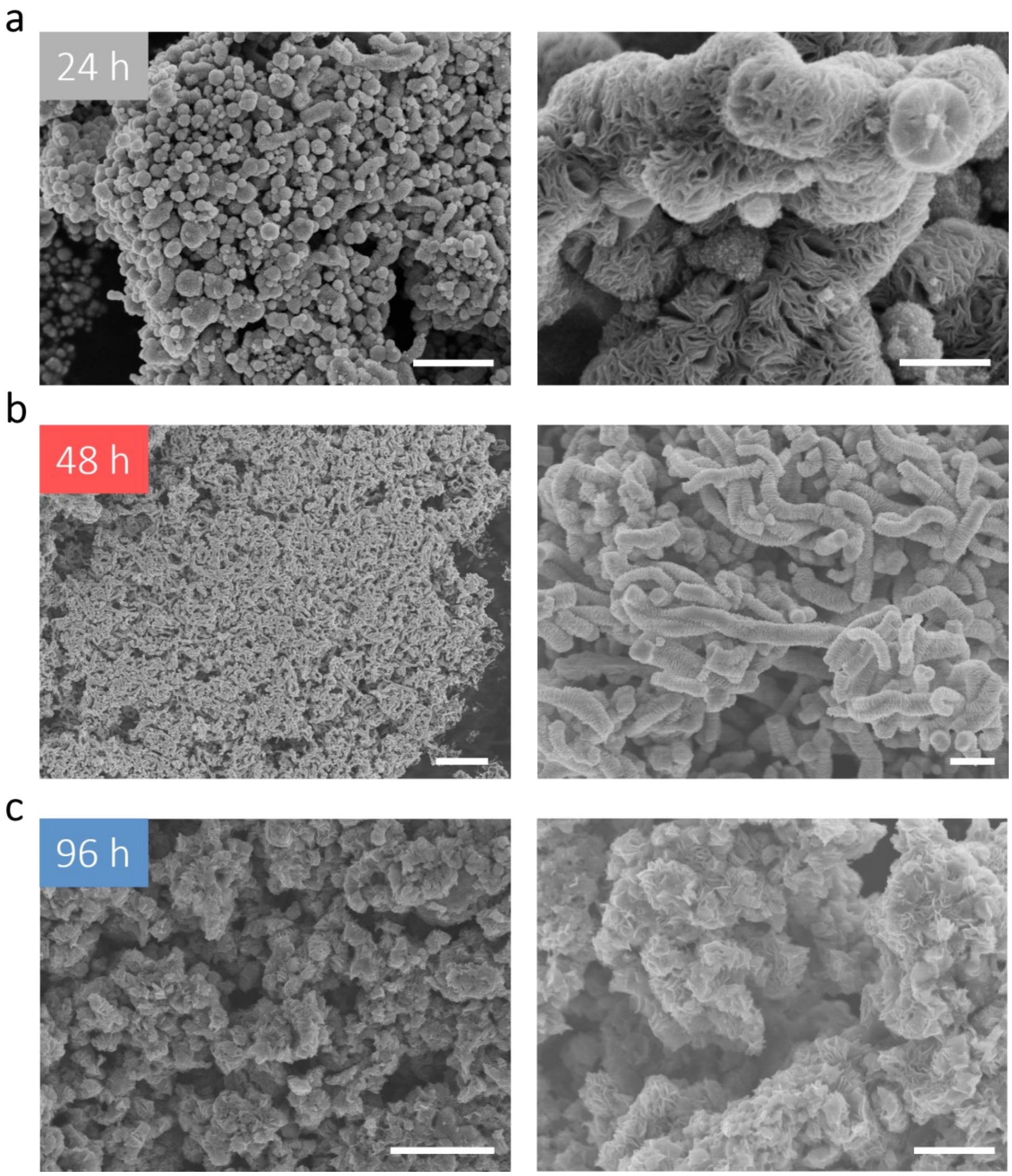

Supplementary Figure 2 | Different magnified SEM images of as-prepared tungsten sulfide samples under different reaction conditions. **a** Reaction time: 24 h. Scale bars represent 2 μm and 500 nm, respectively. **b** Reaction time: 48 h. Scale bars represent 10 μm and 1 μm, respectively. **c** Reaction time: 96 h. Scale bars represent 2 μm and 1 μm, respectively.

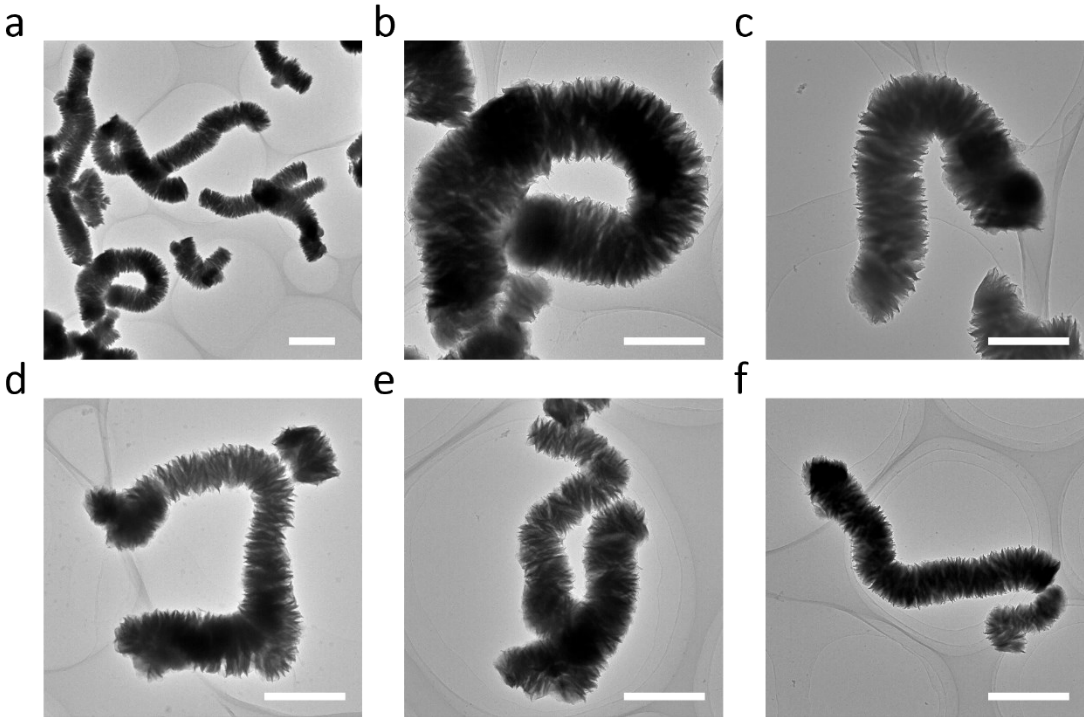

Supplementary Figure 3 | Morphologies of the WS_2 superstructure. **a–f** TEM images of WS_2 superstructure, showing a variety of curling structures. The scale bars for **a** and **b–f** represent 1 μm and 500 nm, respectively.

a b

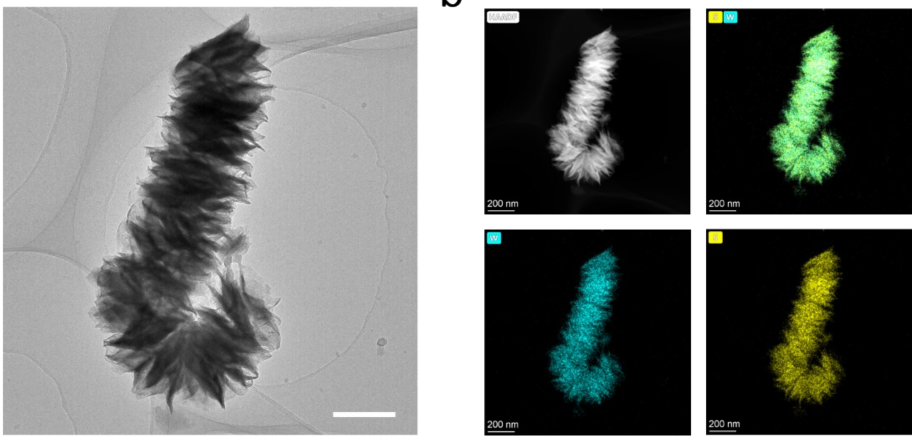

Supplementary Figure 4 | Structure characterization for WS_2 superstructure. **a** HRTEM image of the WS_2 superstructure. **b** HAADF-STEM image and corresponding EDS elemental mapping results of the WS_2 superstructure. Scale bar, 200 nm.

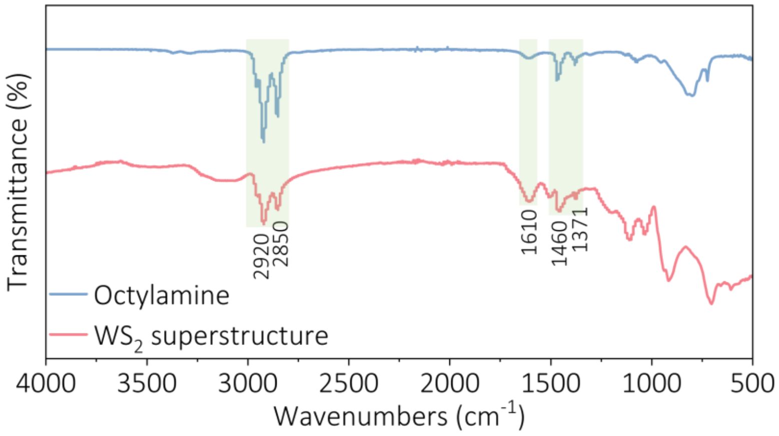

Supplementary Figure 5 | FTIR spectra of the as-prepared WS_2 superstructure and the pure octylamine.

The Fourier transform infrared (FTIR) spectra of the as-prepared sample and the pure octylamine were characterized and presented in Supplementary Fig. 5. The strong peaks at 2920, 2850, 1430, and 1371 cm^{-1} are attributed to the vibration of CH_2 and CH_3 groups, confirms the existence of octylamine molecules in the as-prepared WS_2 superstructure. The strong peaks from $-NH_2$ group at 1610 cm^{-1} in the as-prepared WS_2 superstructure are also observed, which may further suggest the intercalation of octylamine molecules between WS_2 layers[1,2].

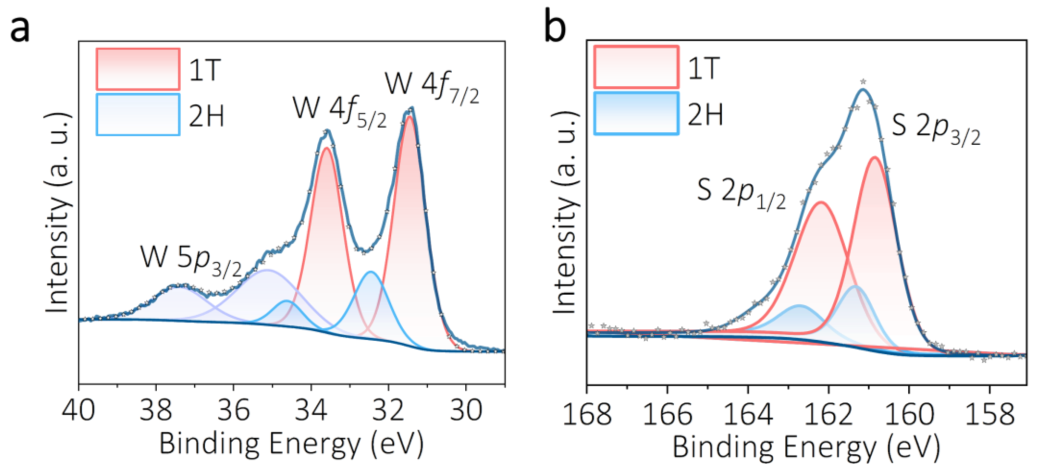

Supplementary Figure 6 | Structure characterization of WS_2 superstructure. **a**, **b** High-resolution XPS spectra of W $4f$ (a) and S $2p$ (b) core level peak regions of the WS_2 superstructure. The fitting red and blue curves represent the contributions of 1T and 2H phases, respectively.

To understand the chemical states and composition of the WS_2 superstructure sample, an X-ray photoelectron spectroscopy (XPS) analysis was conducted. The difference between the measured value and the reference value (284.8eV) of C1s was used as the charged correction value (Δ) to calibrate the binding energy of other elements in the spectrum. Due to the different chemical environment of atom, the electron binding energy of its inner shell will change, which is shown as the peak shift in the spectrogram. The change of the displacement can be used to explain the surface chemical state of the sample or the electronic interaction between the elements on the sample surface. The displacement of the binding energy of all inner electrons is almost the same for atoms with a fixed shell structure. In general, when an element loses an electron, its binding energy shifts toward the high field. The tungsten signal is sensitive to its oxidation state and coordination geometry, thus monitoring the position of the binding energy of the W $4f_{7/2}$ and W $4f_{5/2}$ core level peaks allows one to unambiguously distinguish the distinct W species and can be used to determine the relative ratio of 1T and 2H phases in the WS_2 superstructure[3,4].

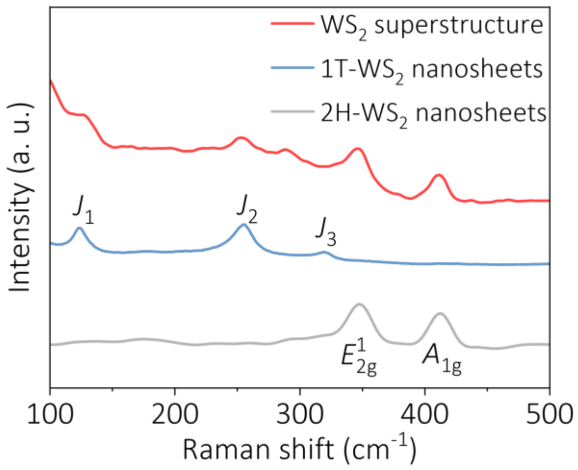

Supplementary Figure 7 | Raman spectra of as-obtained WS_2 superstructure, 1T-WS_2 nanosheets and 2H-WS_2 nanosheets, respectively.

Raman spectroscopy measurements were also performed to further confirm the phase classification. Supplementary Fig. 7 showed comparison of typical Raman spectra of fresh WS_2 superstructure, 1T-WS_2 nanosheets, and 2H-WS_2 nanosheets samples. Two prominent peaks corresponding to the in-plane E^1_{2g} and out-of-plane A_{1g} modes of 2H-WS_2 phase were observed in WS_2 superstructure. The WS_2 superstructure sample exhibited small peaks in the lower frequency region that correspond to the 1T-WS_2 Raman active modes which were not allowed in the 2H-WS_2. These peaks should be attributed to the J_1, J_2, and J_3 vibration modes of S-W-S bonds in 1T-WS_2, suggesting WS_2 exists in hybrid 2H and 1T structures in the as-prepared superstructure[3,4].

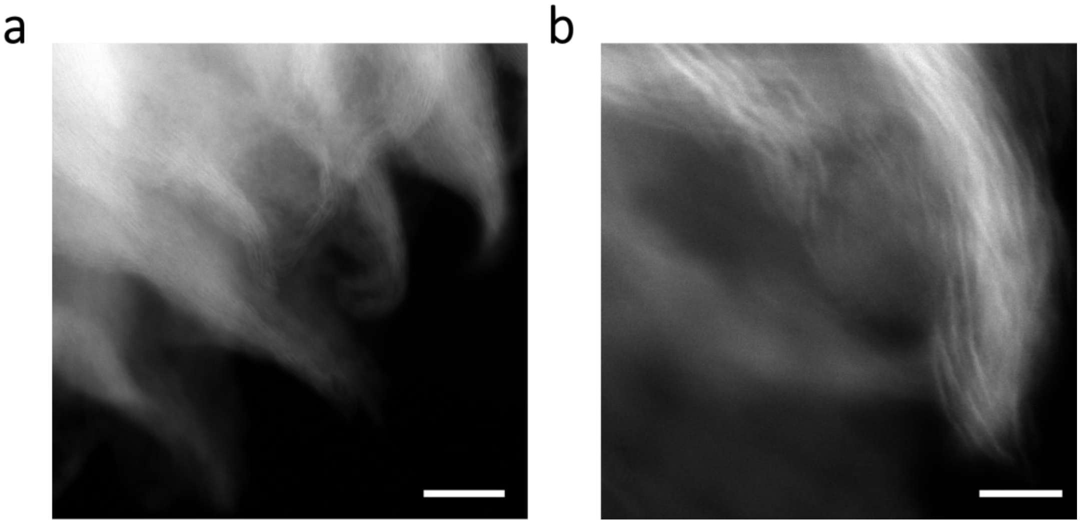

Supplementary Figure 8 | HAADF-STEM images of as-prepared WS_2 superstructure samples at different scales. (**a**) 20 nm, (**b**) 5 nm.

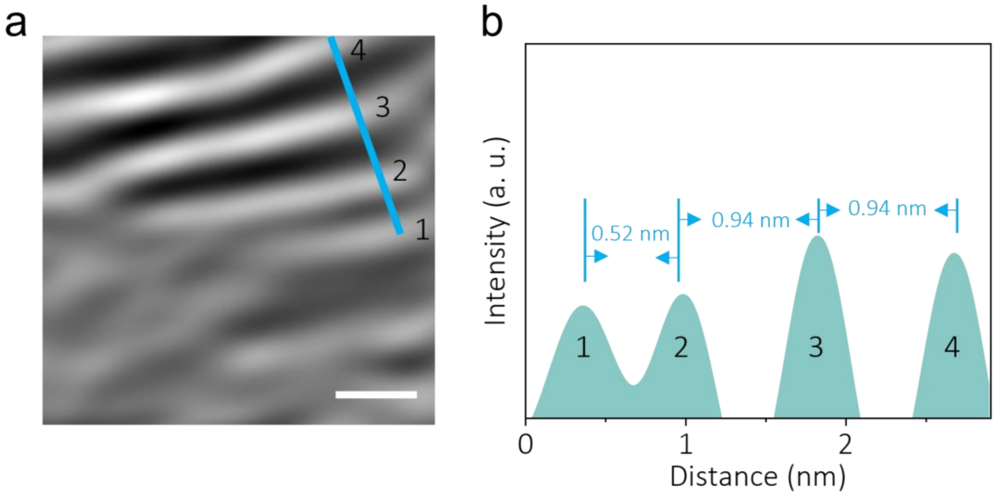

Supplementary Figure 9 | Structural analysis of stepped edge defect structures of WS_2 superstructure. **a** Filtered HRTEM image of the WS_2 superstructure. Scale bar, 1 nm. **b** Line scan of the HRTEM image indicated by the blue line in (**a**), indicating the changed layer spacing.

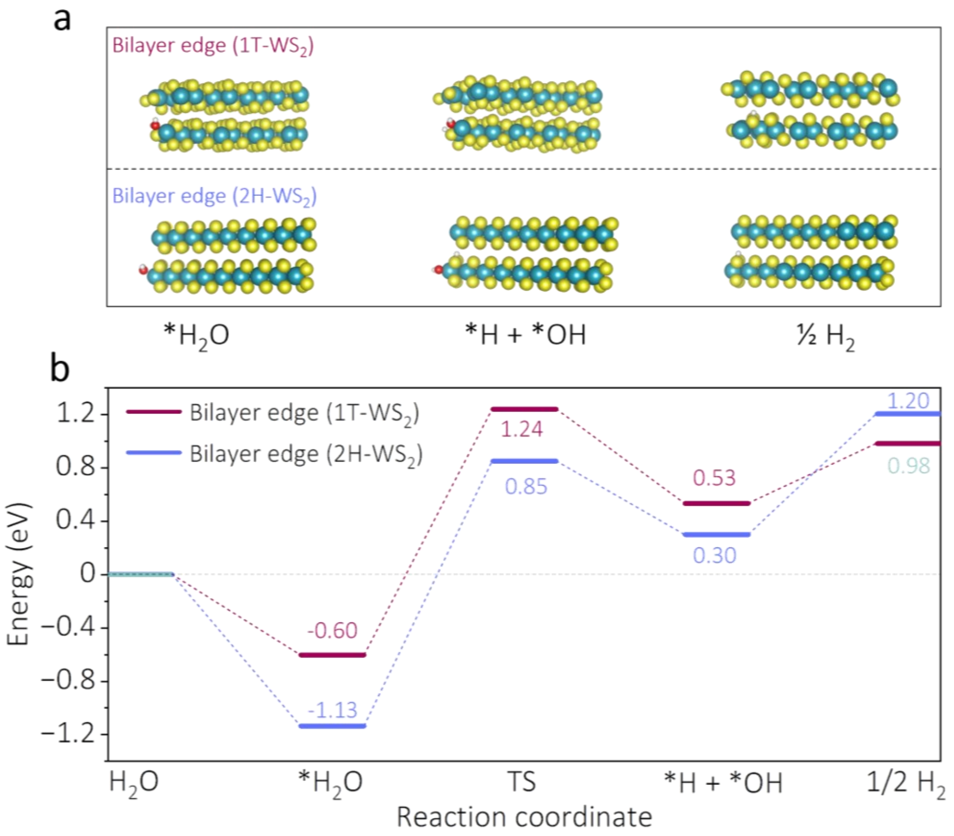

Supplementary Figure 10 | HER mechanism analyses of bilayer edge (1T-WS_2) and bilayer edge (2H-WS_2). **a**, **b** Schematics and free energy diagram for the alkaline HER of bilayer edge (1T-WS_2) and bilayer edge (2H-WS_2). W, S, O, and H atoms are represented by cyan, yellow, red, and white spheres, respectively.

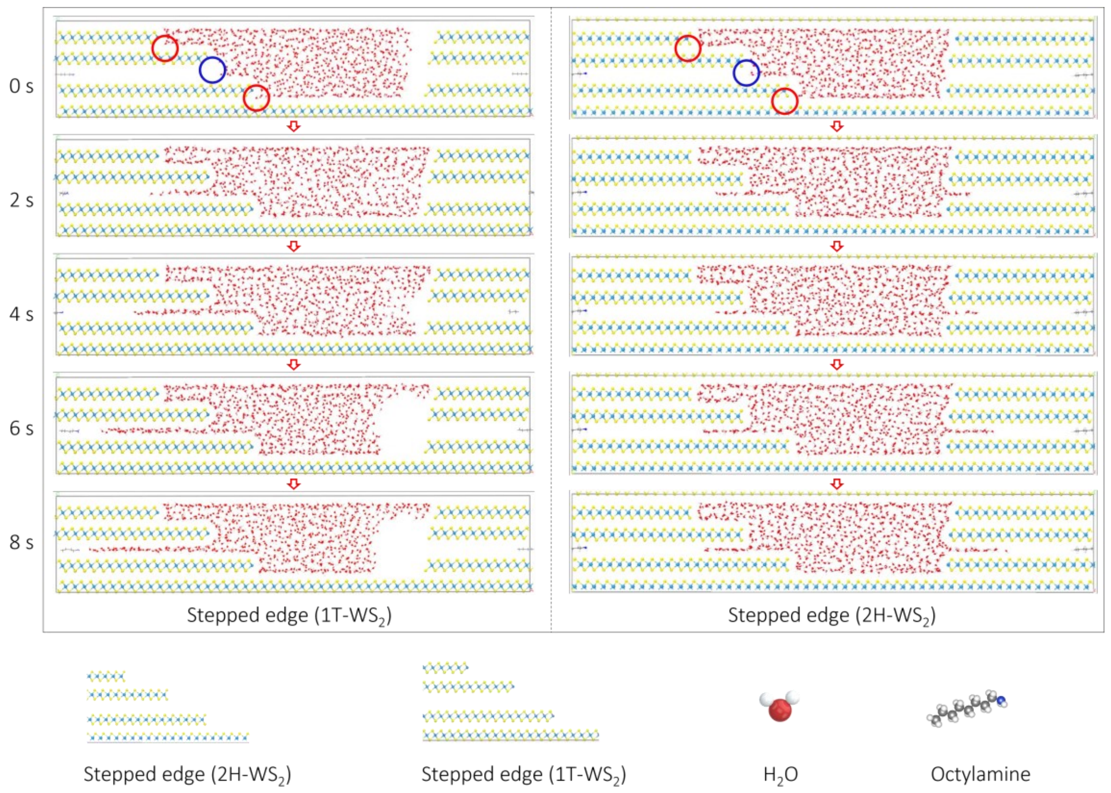

Supplementary Figure 11 | Multiple snapshots (time sequences) of water molecules on the stepped edge (1T-WS_2) and stepped edge (2H-WS_2) structures.

Molecular dynamics (MD) simulation is a powerful tool for the intensive study of electrocatalytic HER processes by employing theoretical and computational methods to simulate molecular motions at the microscopic level[5,6]. We have used MD simulation to investigate the effect of a stepped edge defect structure with enlarged interlayer spacing on the transport of water molecules within the electrochemical reaction system. In the MD simulations, we employed the projector augmented wave potential for nuclei-electron interactions and the Γ point for Brillouin zone sampling. The time step for the MD simulation is 0.5 fs and the canonical ensemble condition (NVT) was imposed by a Nose-Hoover thermostat with a target temperature of 300 K. The snapshots of MD simulation results were prepared by visual molecular dynamic

(VMD)[7]. Based on the observed structural features in Supplementary Fig. 9, we constructed the edge-stepped defect structural models using the Vienna Ab initio Simulation Package (VASP). The relative density distribution of water molecules in Supplementary Fig. 11 indicated that it was difficult for water molecules to enter the interlayer of stepped edge WS_2 with intrinsic interlayer spacing (marked by red circles). In sharp contrast, water molecules exhibit rapid diffusion into the interlayers of the stepped edge WS_2 model featuring enlarged interlayer spacing (marked by dark blue circles). Moreover, we found that water molecules have a faster diffusion rate in the interlayer of stepped edge (1T-WS_2) than stepped edge (2H-WS_2). Specifically, when the diffusion time reaches 8 s, the water molecules have diffused to the other end of stepped edge (1T-WS_2) model in comparison with the stepped edge (2H-WS_2) model. These results suggest that the stepped edge defect structure with enlarged layer spacing enhances the transport of water molecules at the electrode surface interface, contributing to the acceleration of charge transfer between the active center and water molecules as well as the migration of intermediate species, which has the potential to enhance the alkaline HER kinetics.

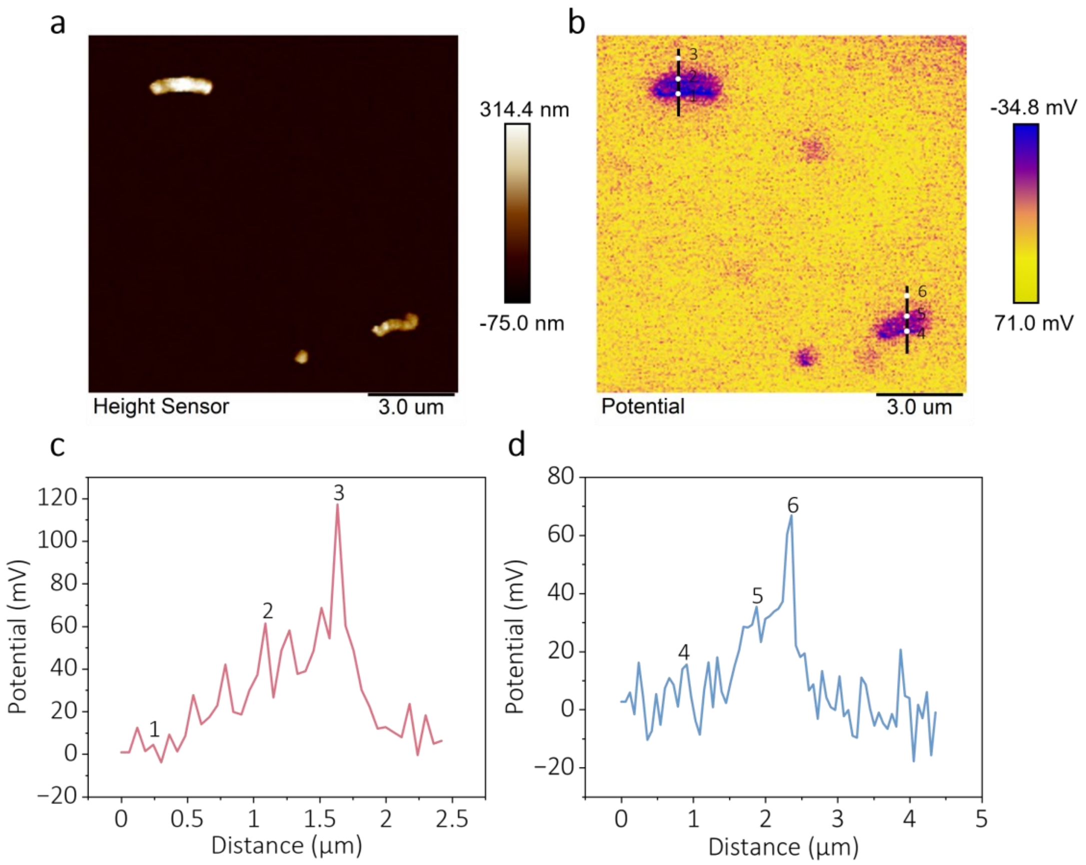

Supplementary Figure 12 | AFM characterization of WS_2 superstructure. **a** AFM image of single WS_2 superstructure. **b–d** Surface potential of WS_2 superstructure detected with KPFM.

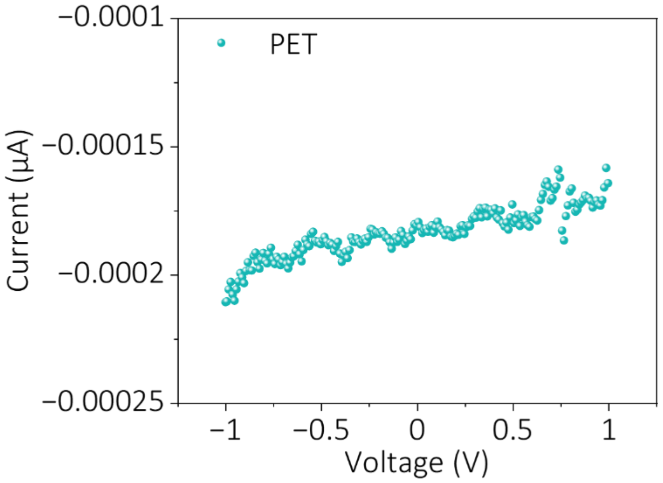

Supplementary Figure 13 | Current-voltage curve for the PET substrate.

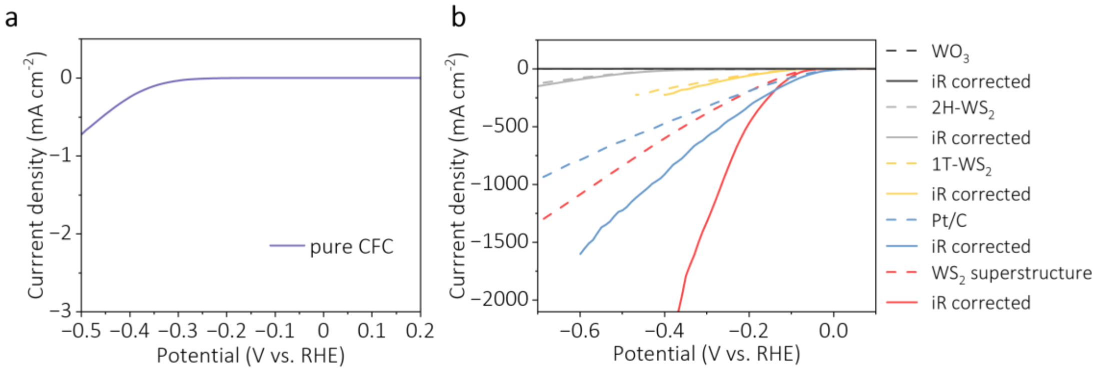

Supplementary Figure 14 | HER performance of different samples. **a** LSV polarization curve of bare CFC (1 cm^2), indicating that the bare CFC exhibits negligible electrocatalytic performance for the HER. **b** LSV polarization curves of all samples in 1 M KOH (without and after iR-corrected dates).

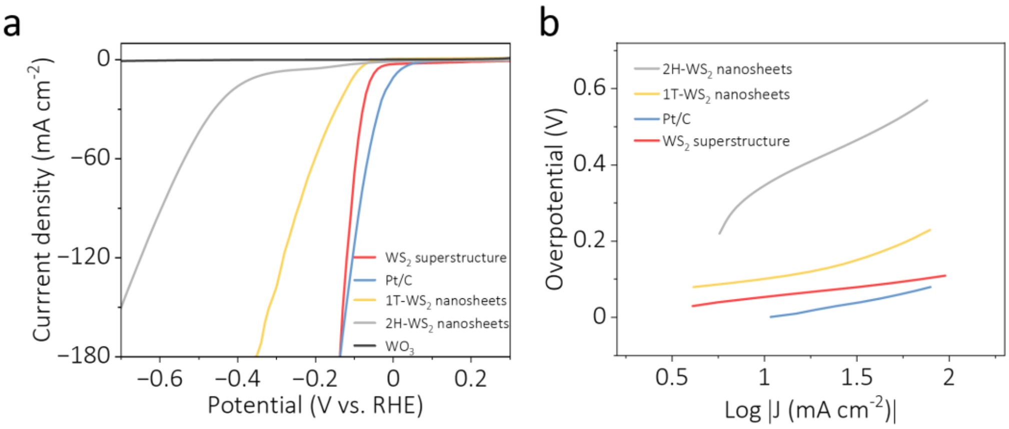

Supplementary Figure 15 | Electrochemical HER performance of various samples. **a** Polarization curves at low current density. **b** Corresponding tafel slopes derived form (**a**).

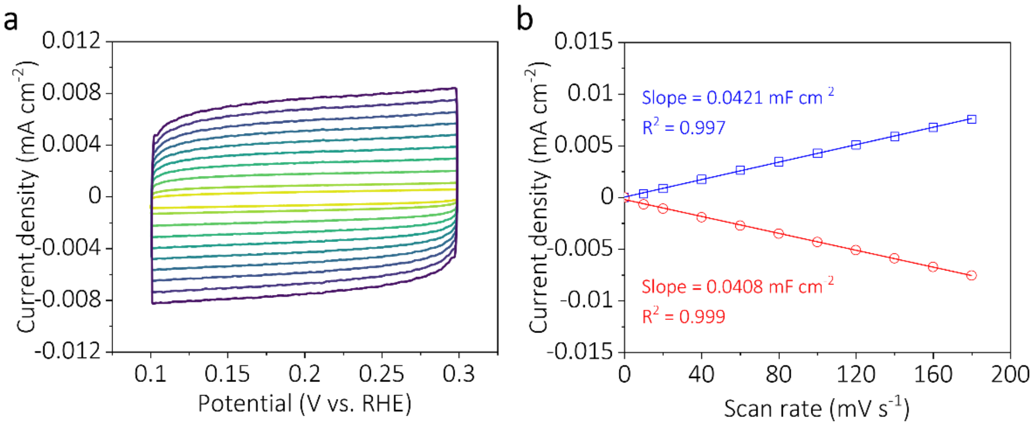

Supplementary Figure 16 | Double-layer capacitance measurements for determining electrochemically-active surface area for the substrate material (bare CFC) from voltammetry. **a** Cyclic voltammograms were measured in a non-Faradaic region of the voltammogram at the following scan rate: 10, 20, 40, 60, 80, 100, 120, 140, 160, and 180 mV s^{-1}. The working electrode was held at each potential vertex for 10 s before the beginning the next sweep. All current is assumed to be due to capacitive charging. **b** The cathodic (○) and anodic (□) charging currents measured at 0.20 V vs. RHE plotted as a function of scan rate.

The determined double-layer capacitance of the system is taken as the average of the absolute value of the slope of the linear fits to the data—here it is 0.0415 mF cm^{-2}.

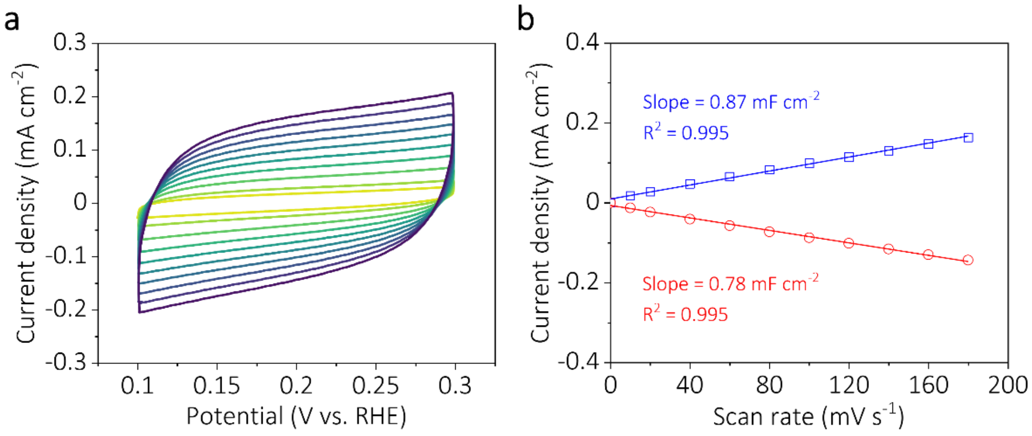

Supplementary Figure 17 | Double-layer capacitance measurements for determining electrochemically-active surface area for 2H-WS_2 sample from voltammetry. **a** Cyclic voltammograms were measured in a non-Faradaic region of the voltammogram at the following scan rate: 10, 20, 40, 60, 80, 100, 120, 140, 160, and 180 mV s^{-1}. The working electrode was held at each potential vertex for 10 s before the beginning the next sweep. All current is assumed to be due to capacitive charging. **b** The cathodic (○) and anodic (□) charging currents measured at 0.20 V vs. RHE plotted as a function of scan rate.

The determined double-layer capacitance of the system is taken as the average of the absolute value of the slope of the linear fits to the data—here it is 0.825 mF cm^{-2}. Assuming a general specific capacitance of 60 mF cm^{-2} gives an electrochemically active surface area of 13.7 cm^2_{ECSA} from this measurement.

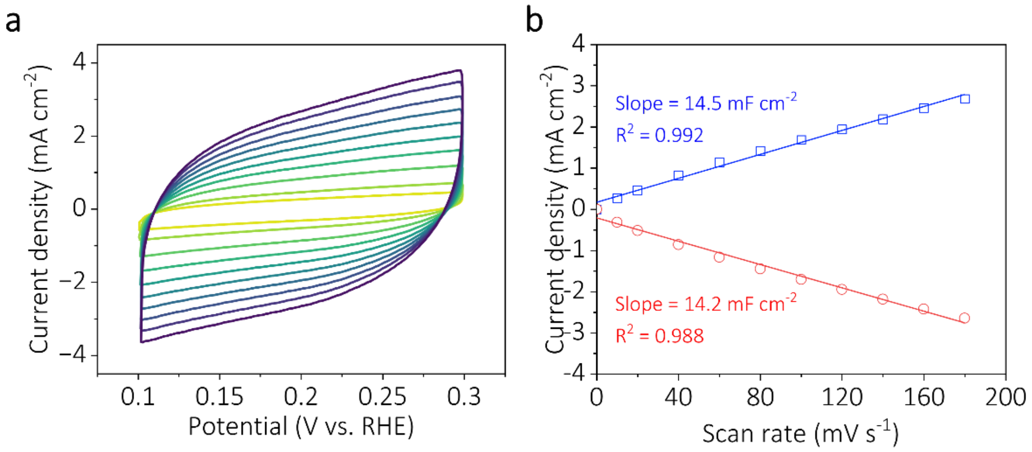

Supplementary Figure 18 | Double-layer capacitance measurements for determining electrochemically-active surface area for 1T-WS_2 sample from voltammetry. **a** Cyclic voltammograms were measured in a non-Faradaic region of the voltammogram at the following scan rate: 10, 20, 40, 60, 80, 100, 120, 140, 160, and 180 mV s^{-1}. The working electrode was held at each potential vertex for 10 s before the beginning the next sweep. All current is assumed to be due to capacitive charging. **b** The cathodic (○) and anodic (□) charging currents measured at 0.20 V vs. RHE plotted as a function of scan rate.

The determined double-layer capacitance of the system is taken as the average of the absolute value of the slope of the linear fits to the data—here it is 14.35 mF cm^{-2}. Assuming a general specific capacitance of 60 mF cm^{-2} gives an electrochemically active surface area of 239.2 cm^2_{ECSA} from this measurement.

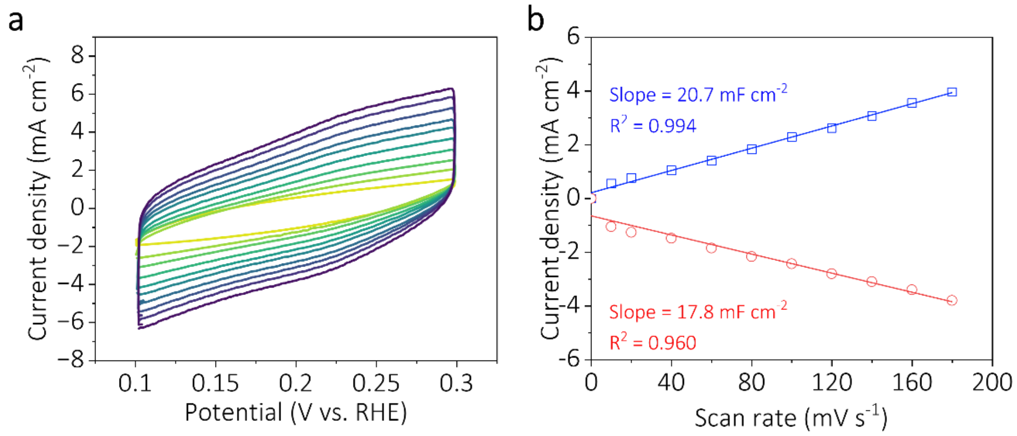

Supplementary Figure 19 | Double-layer capacitance measurements for determining electrochemically-active surface area for WS_2 superstructure sample from voltammetry. **a** Cyclic voltammograms were measured in a non-Faradaic region of the voltammogram at the following scan rate: 10, 20, 40, 60, 80, 100, 120, 140, 160, and 180 mV s^{-1}. The working electrode was held at each potential vertex for 10 s before the beginning the next sweep. All current is assumed to be due to capacitive charging. **b** The cathodic (○) and anodic (□) charging currents measured at 0.20 V vs. RHE plotted as a function of scan rate.

The determined double-layer capacitance of the system is taken as the average of the absolute value of the slope of the linear fits to the data—here it is 19.25 mF cm^{-2}. Assuming a general specific capacitance of 60 mF cm^{-2} gives an electrochemically active surface area of 320.8 cm^2_{ECSA} from this measurement.

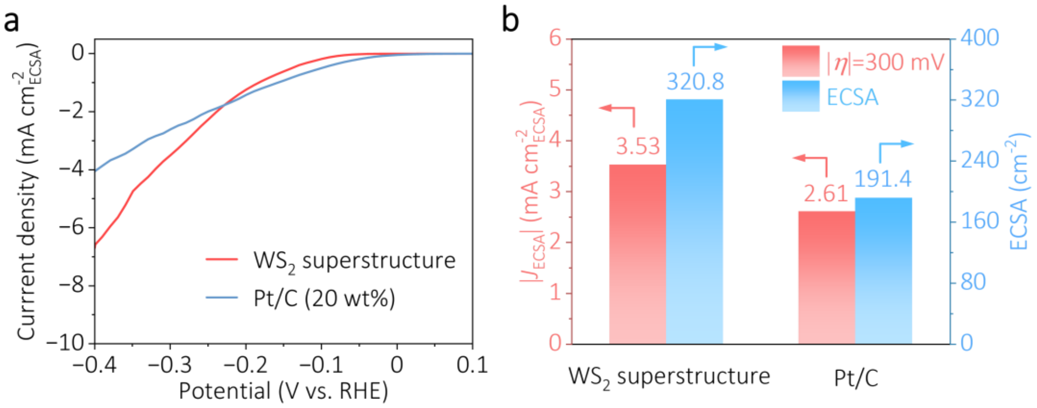

Supplementary Figure 20 | Comparison of intrinsic activity of different samples. **a** HER activity normalized for the electrochemical active surface area (ECSA). **b** Comparison of the ECSA and J_{ECSA} (at −0.3 V vs. RHE) of WS_2 superstructure and commercial Pt/C (20 wt%).

As shown in Supplementary Fig. 20, the ECSA-normalized current density demonstrated that the WS_2 superstructure still reveals substantially higher HER current density than that of Pt/C at a high overpotential (> −228 mV vs. RHE). The TOF results in Supplementary Table 2 demonstrated that the TOF (at −200 mV vs. RHE) of WS_2 superstructure is 4.011 s^{-1}, higher than that of commercial Pt/C (2.670 s^{-1}). These results demonstrated that the WS_2 superstructure catalysts exhibit a superior HER performance relative to Pt/C (20 wt%).

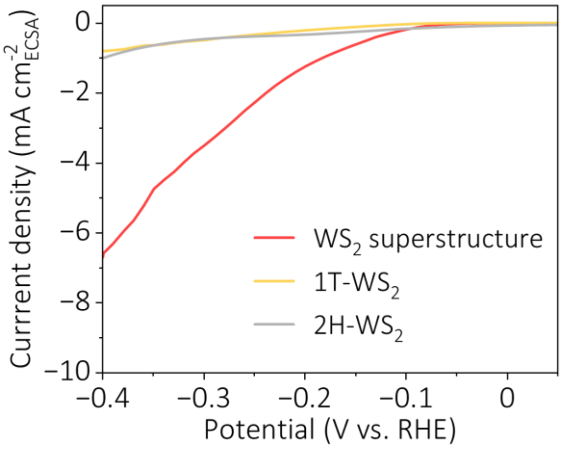

Supplementary Figure 21 | HER activity normalized for the electrochemical active surface area (ECSA).

To further demonstrate the enhanced intrinsic activity of WS_2 superstructure, the polarization curves were normalized to electrochemically active surface area (ECSA), which was derived from the double-layer capacitance (C_{dl}, Supplementary Figs. 16–19). As shown in Supplementary Fig. 21, WS_2 superstructure still reveals substantially higher HER current density than that of 1T-WS_2 and 2H-WS_2 at the same overpotential under the same measurement conditions.

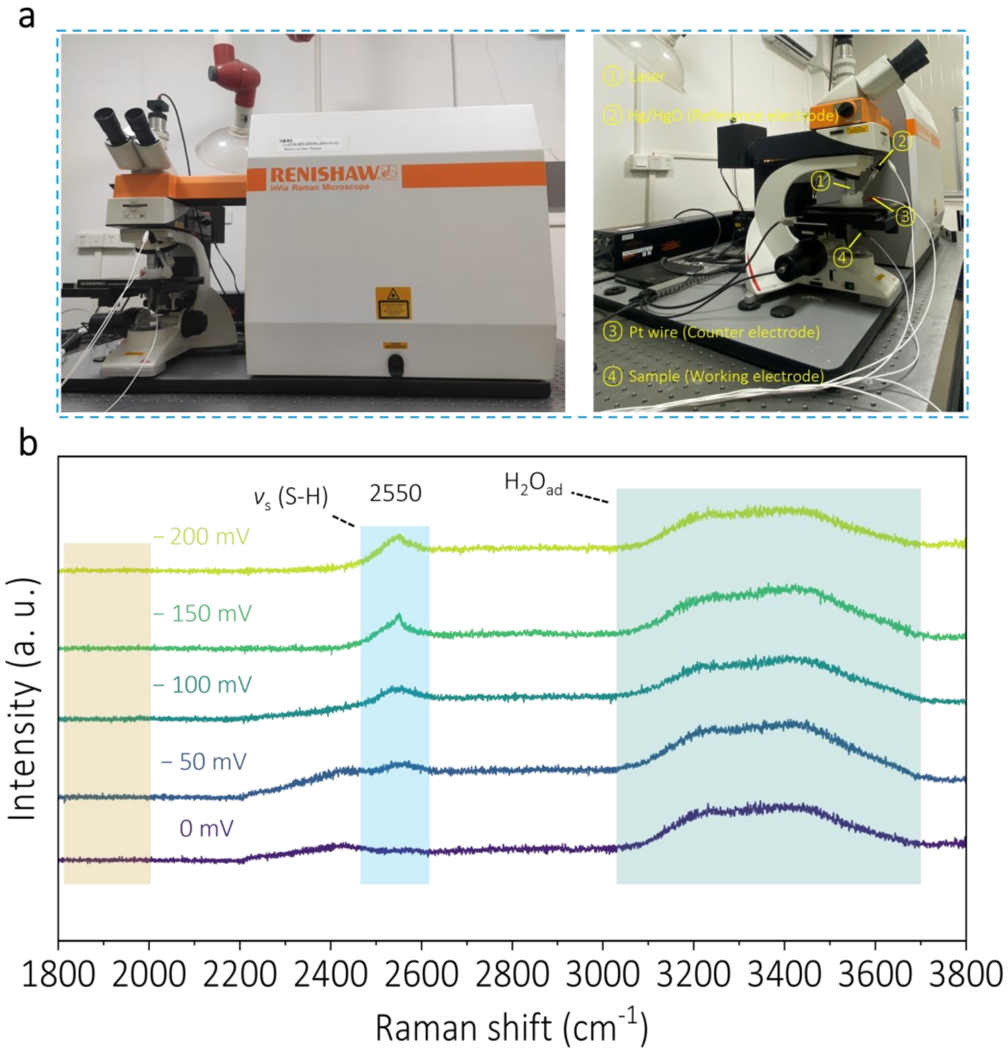

Supplementary Figure 22 | In-situ Raman spectra of catalysts evolution during the HER processes. **a** Schematic illustration of the in-situ Raman spectroscopy experimental set-up. **b** In-situ SERS spectra of the WS_2 superstructure catalyst at various potentials (vs. RHE) under HER conditions in 1.0 M KOH.

Capturing direct spectroscopic evidence of intermediates produced during catalytic processes is key to unraveling the origin of HER activity enhancement[8]. Surface-enhanced Raman spectroscopy (SERS) can provide surface-sensitive as well as chemical bond specific signals at the atomic level, making it a powerful fingerprint spectroscopy which can in-situ identify the active sites as well as the surface reaction intermediates during catalytic processes[9,10]. To reveal how the defect structure of WS_2 improves the overall activity of the WS_2 superstructure, in-situ SERS was used to

monitor the HER process in 1.0 M KOH electrolyte (Supplementary Fig. 22a).

Specifically relevant to the alkaline solution of the HER, where proton generation through water decomposition is pivotal, the assessment of water adsorption capacity assumes critical significance as an indicator of catalytic activity[4]. As shown in Supplementary Fig. 22b, the broad peak spanning from 3000 to 3700 cm^{-1} corresponds to the adsorbed water peaks in the WS_2 superstructure sample, demonstrating its excellent water adsorption capacity[11]. When the potential reaches −50 mV, an obvious Raman peak located at ca. 2550 cm^{-1} appears and its intensity increases as the potential further decreases to −200 mV. The band detected at 2550 cm^{-1} is ascribed to the stretching vibration of S-H bond, *v*(S-H)[12–14], indicating that the H atom is directly bonded to the sulfur atom of WS_2 superstructure during the HER. Importantly, it is worth mentioning that the vibrational signals, *v*(W-H)[15], between 1831 and 1993 cm^{-1} have not been observed in the in-situ SERS measurements (Supplementary Fig. 22). Based on detailed fingerprint information mentioned above, it is concluded that the sulfur atom of WS_2 is the catalytic active site for the HER. Albeit different from our WS_2 superstructure, the sulfur atoms in amorphous MoS_x[16] and MoS_2 nanosheets[13] were also confirmed to be the active sites for HER.

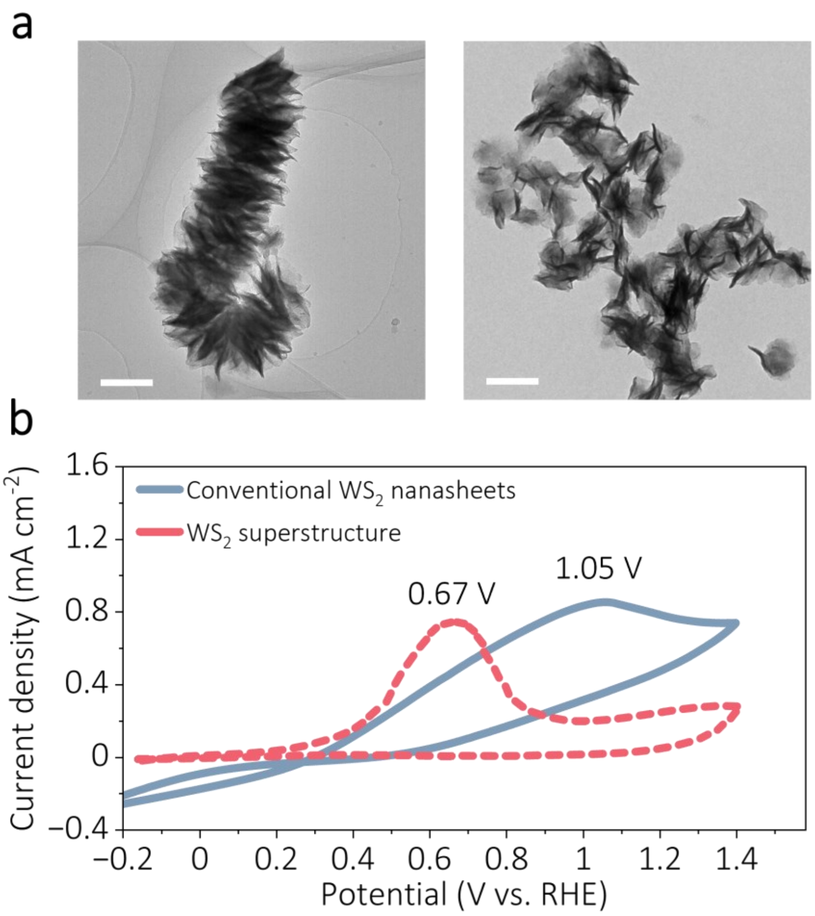

Supplementary Figure 23 | Electrochemical analysis of the edge sites in different samples. **a** TEM images of WS_2 superstructure (left) and conventional WS_2 nanosheets (right). All the scale bars in (a) are 200 nm. The conventional WS_2 nanosheets were synthesized according to a previous report[3] for comparison. **b** Irreversible electrochemical oxidation cyclic voltammetry curves. Irreversible electrochemical oxidation of WS_2 superstructure and conventional WS_2 nanosheets in 0.5 M H_2SO_4 at a scan rate of 60 mV s^{-1}.

Besides of the HRTEM images (Fig. 2f and Supplementary Fig. 9), the irreversible electrochemical oxidation of metal sulfides was also investigated as a measure of their edge sites. Irreversible electrochemical oxidations were performed on both samples (WS_2 superstructure and conventional WS_2 nanosheets, as shown in Supplementary Fig.

23a) using 0.5 M H_2SO_4 as the electrolyte at a scan rate of 60 mV s^{-1}. Supplementary Fig. 23b shows the cyclic voltammetry (CV) curves of the WS_2 superstructure and conventional WS_2 nanosheets. A peak centered at ~1.05 V (vs. RHE) in the CV curve of conventional WS_2 nanosheets can be ascribed to the oxidation of basal planes of WS_2[17]. In contrast, one apparent oxidation peak centered at ~0.67 V vs. RHE was observed in the CV of the WS_2 superstructure, possibly due to its edge-rich (including both sheet edges and stepped defect edges) features, as edges of WS_2 nanostructures are expected to be more readily oxidized than does the basal plane[16,18]. Therefore, the edge-rich characteristic of the WS_2 superstructure is confirmed again.

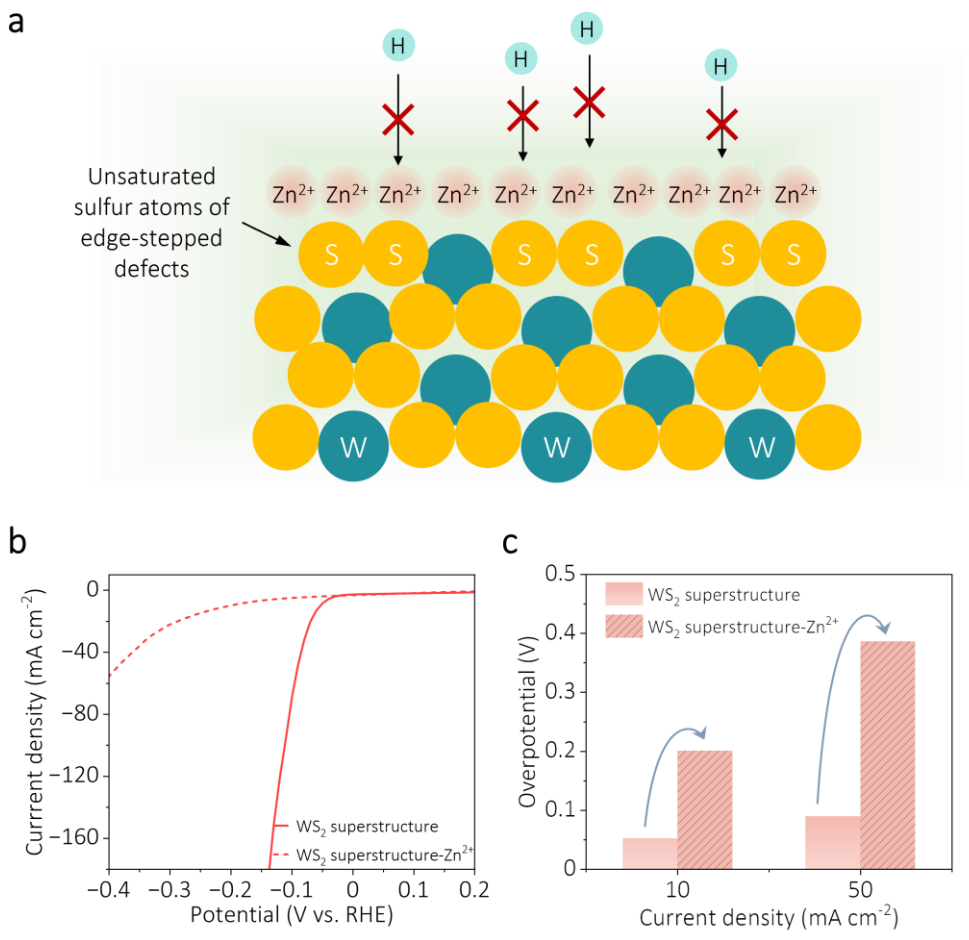

Supplementary Figure 24 | The poisoning experiment of the real active sites. **a** Schematic diagram of the active site blocking mechanism. **b**, **c** HER polarization curves (**b**) and the required overpotential at 10 and 50 mA cm^{-2} (**c**) of WS_2 superstructure before and after soaking in 1.0 mM $Zn(NO_3)_2$ solution for 30 s.

To further verify the significance of S active sites for HER, zinc nitrate ($Zn(NO_3)_2$) was employed in the poisoning experiment to block the S sites, as depicted in Supplementary Fig. 24a, according to the previous report[12,19]. As shown in Supplementary Fig. 24b, the HER activity of the WS_2 superstructure decreased significantly after being soaked in 1.0 mM $Zn(NO_3)_2$ aqueous solution for 30 s. The

poisoning of active S sites resulted in an increase of overpotential from 52 mV to 201 mV at 10 mA cm^{-2} and 89 to 386 mV at 50 mA cm^{-2} (Supplementary Fig. 24c). We can conclude from the poison experiments that edge-stepped defects with abundant unsaturated sulfur atoms play an important role in determining the HER property of the WS_2 superstructure electrocatalyst.

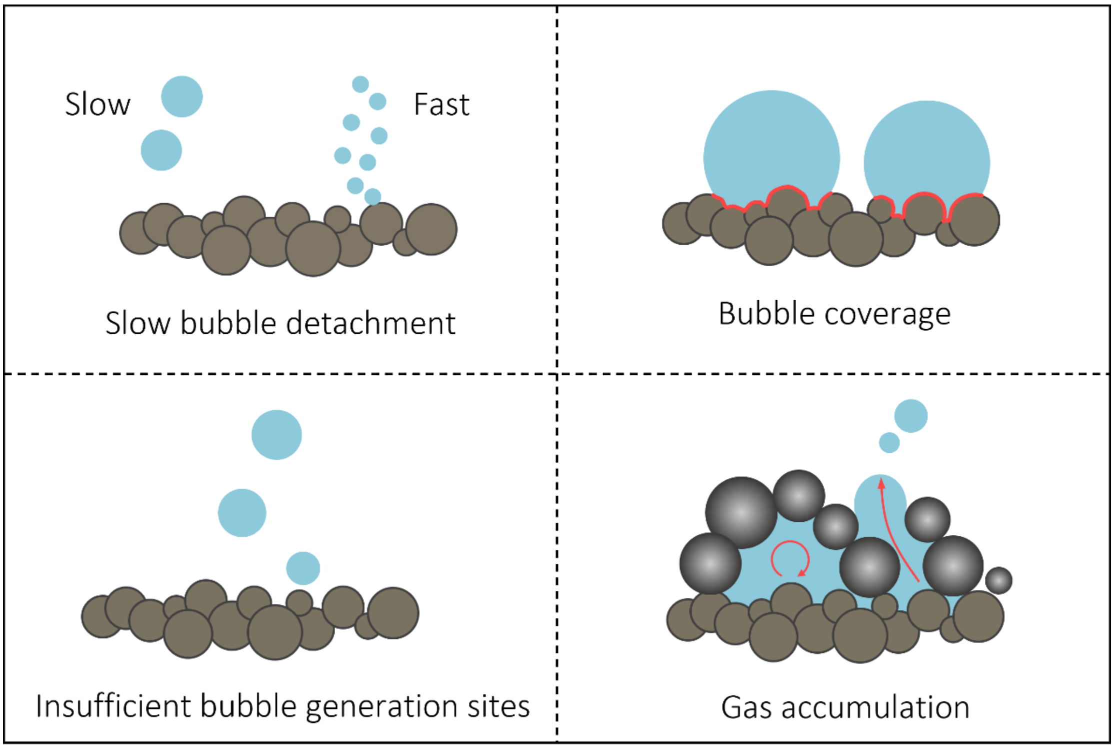

Supplementary Figure 25 | Bubble issues of electrocatalyst under high current density operation.

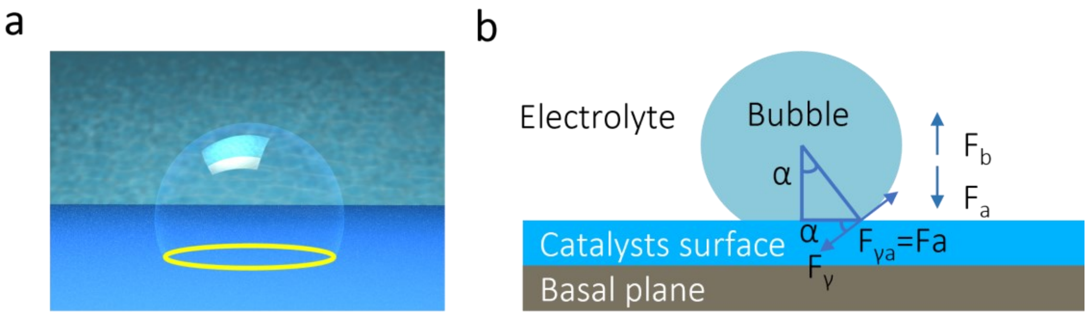

Supplementary Figure 26 | Force analysis of bubble on the surface of ideal electrode. **a** Continuous three-phase (solid-liquid-gas) contact line (TPCL) (yellow line) at the electrode surface. **b** Stress analysis of one single bubble on the electrode surface.

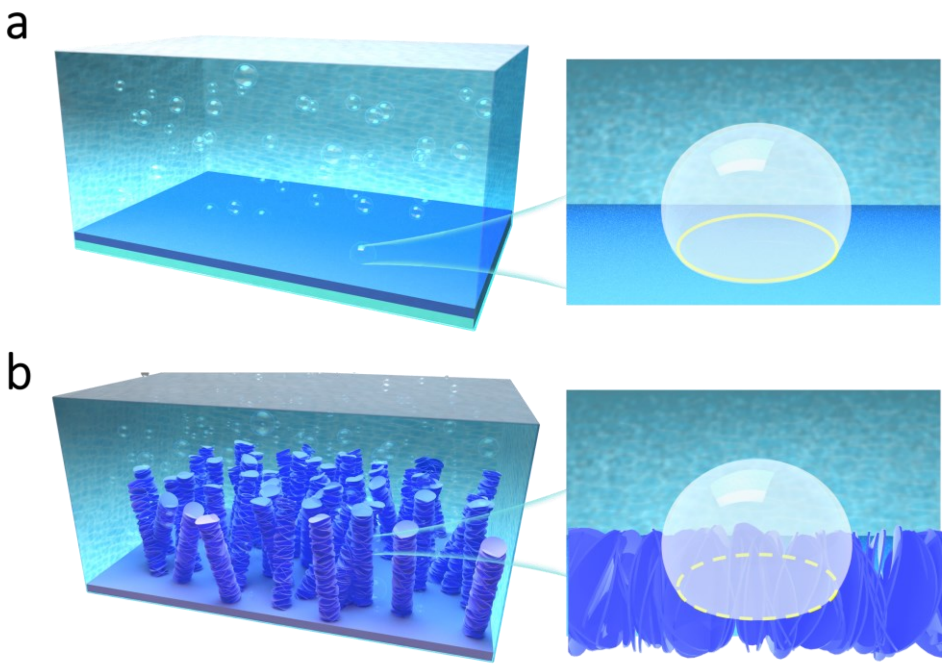

Supplementary Figure 27 | Schematic illustration of how the electrode surface morphology affecting the bubble contacts and release. Continuous and discontinuous TPCL (yellow line) at flat (**a**) and superstructure electrode surface (**b**).

Gas-involving electrochemical reactions, including gas-evolution reactions (GERs) and gas-consumption reactions (GCRs), are essential components of the energy conversion processes. Generally, GERs include hydrogen evolution reaction (HER), oxygen evolution reaction (OER), hydrazine oxidation reaction (HzOR with N_2 as product), and chlorine evolution reaction (ClER). In water splitting industry, a great number of gas bubbles will generate on the electrode surface as large current densities are usually required. In this case, if the electrode shows strong adhesion to gas bubbles, a large number of gas bubbles will gather around the surface and block the active sites and the diffusion of electrolyte, resulting in huge reaction resistance[20–22], as shown in

Fig. R27.

Since the diameters of bubbles released underwater are correlated with the interaction at the three-phase (solid-liquid-gas) contact line (TPCL, as shown in Supplementary Fig. 26a), the solid electrode surface intended for promoting the gas evolution in GERs should make the gas bubbles smaller for faster bubble release[20]. The simplified stress analysis on an individual bubble at the electrode surface indicated that two main forces governed the bubble detachment (as shown in Supplementary Fig. 26b): buoyant force (F_b) pointing upward and adhesion force (F_a) pointing downward. Thus, the threshold to drive the bubble off the surface can be reached as the buoyant force balances with the adhesion force. The threshold releasing radius is proportional to the root of surface tension ($\sqrt{\gamma}$) and sine function of bubble contact angle (sin α).

$$r \propto \sqrt{\gamma}\ \sin\alpha$$

The adhesion force of a given course surface (F_{a*}) is determined by the area fraction of the solid on the surface (f_s).

$$F_{a*} = f_s F_a$$

where α, r, and γ represent the bubble contact angle, radius of the bubble and the surface tension at the TPCL, respectively. Moreover, F_{a*} is the adhesion force on rough solid surface.

If the electrode surface is a relatively flat surface, the TPCL should be a continuous circle (Supplementary Fig. 27a) and each point of the circle generates the adhesion force. However, the superstructure could break the circle into discontinuous dots where the gaps between isolated standing units are filled with liquid (Supplementary Fig. 27b),

named the “cutting edge effect”, resulting in much smaller bubble adhesion force. Therefore, the bubbles on the superstructure exhibit lower adhesion forces and smaller detachment volumes than flat ones; in other words, the superstructure electrodes possess a superior ability to accelerate gas evolution behavior and improve electrocatalytic performance at high reaction rates.

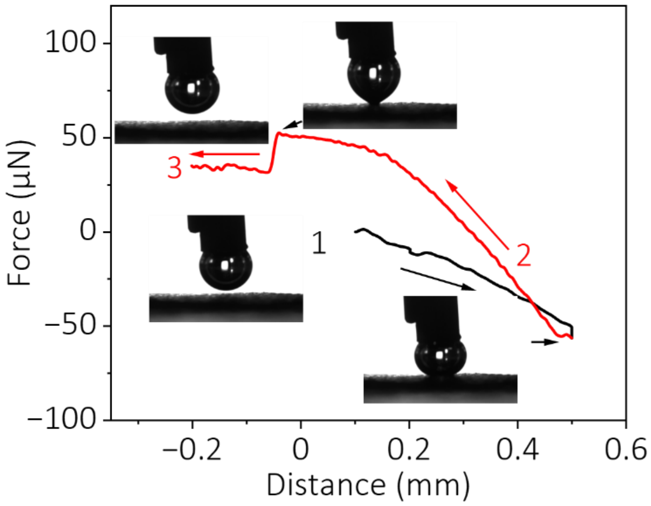

Supplementary Figure 28 | Adhesive force measurement of the bubble on the 1T-WS_2 electrode surface. Insets show the bubble states during the corresponding adhesive force measurement processes.

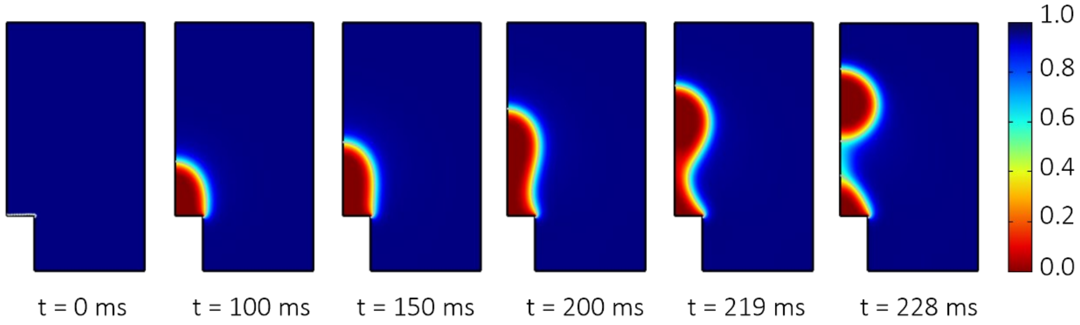

Supplementary Figure 29 | Hydrodynamic simulation of the complete bubble formation process on the hydrophobic surface.

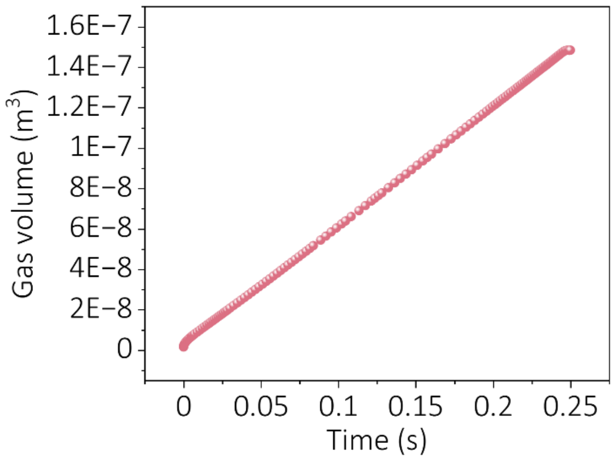

Supplementary Figure 30 | Hydrogen volume fraction plotted against time in finite element simulation.

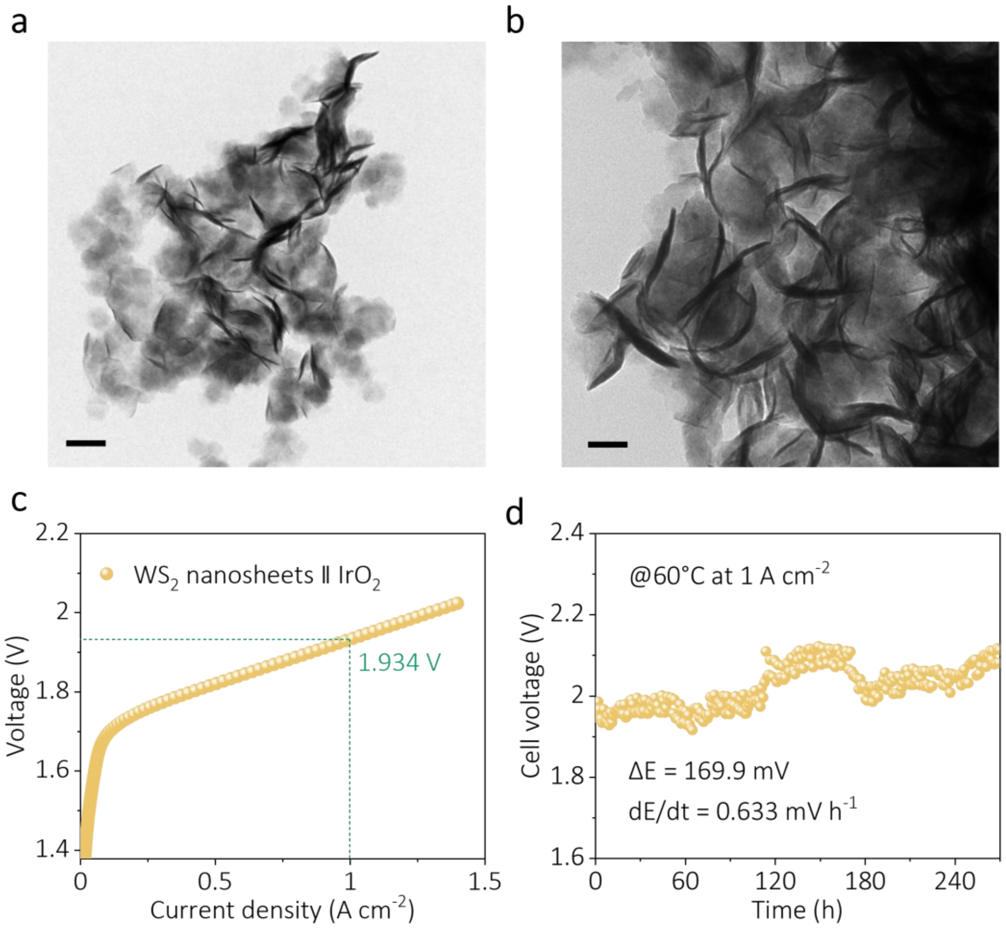

Supplementary Figure 31 | AEM electrolyser performance using conventional WS_2 nanosheets as catalyst. **a**, **b** TEM images of conventional WS_2 nanosheets. **c** Polarization curve of the AEM electrolyser. **d** Durability test of the AEM electrolyser using conventional WS_2 nanosheets at 1 A cm^{-2}.

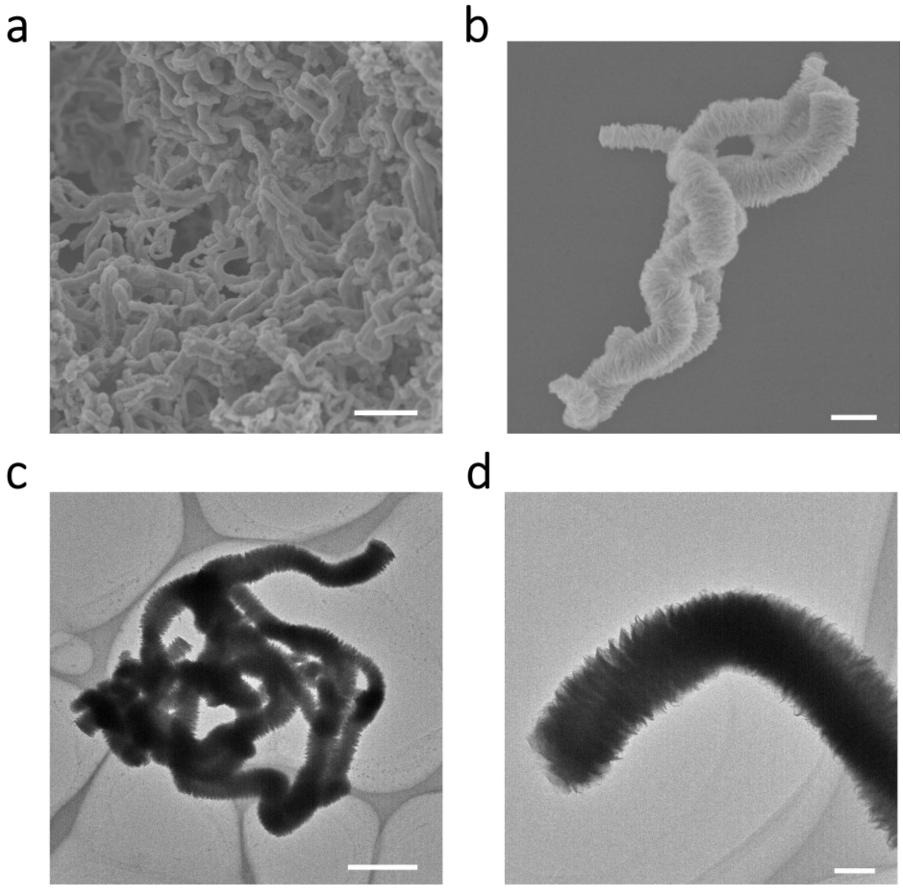

Supplementary Figure 32 | Morphology characterization of the WS_2 superstructure catalyst after 1000 h AEM electrolyser operation. **a**, **b** SEM images. Scale bars represent 2 μm and 500 nm, respectively. **c**, **d** TEM images. Scale bars represent 1 μm and 200 nm, respectively.

The morphology of WS_2 superstructure catalyst after long-term operation in a AEM electrolyser was measured using SEM and TEM, as shown in Supplementary Fig. 32. SEM and TEM images reveal that the WS_2 superstructure still maintains the initial morphology after the operation in the AEM electrolyser.

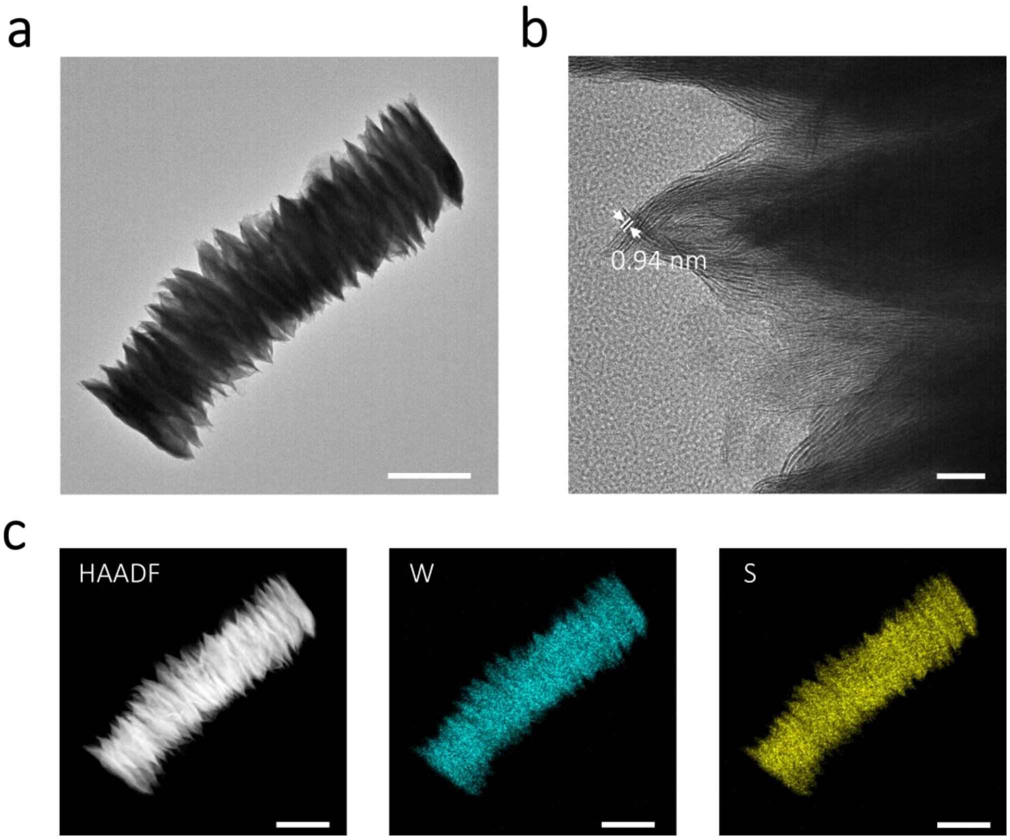

Supplementary Figure 33 | TEM characterization of the WS_2 superstructure catalyst after 1000 h AEM electrolyser operation. **a** TEM image. **b** HRTEM image. **c** elemental mapping images of W and S elements.

Additional TEM experiments were performed to further characterize the WS_2 superstructure catalyst after 1000 h operation in the AEM electrolyser (Supplementary Fig. 33). The post-HER catalyst (WS_2 superstructure) exhibits similar infrastructure to its starting appearance, typical of caterpillar-like morphology assembly with staggered nanosheets with an interlayer spacing of 0.94 nm (Supplementary Fig. 33a, b). Moreover, the EDS elemental mappings reveal that Ir, Ta, Tm, and O elements are homogeneously distributed (Supplementary Fig. 33c). These results further confirm that the WS_2 superstructure has excellent stability and thus is highly promising for long-term alkaline HER operation in the practical AEM electrolyser.

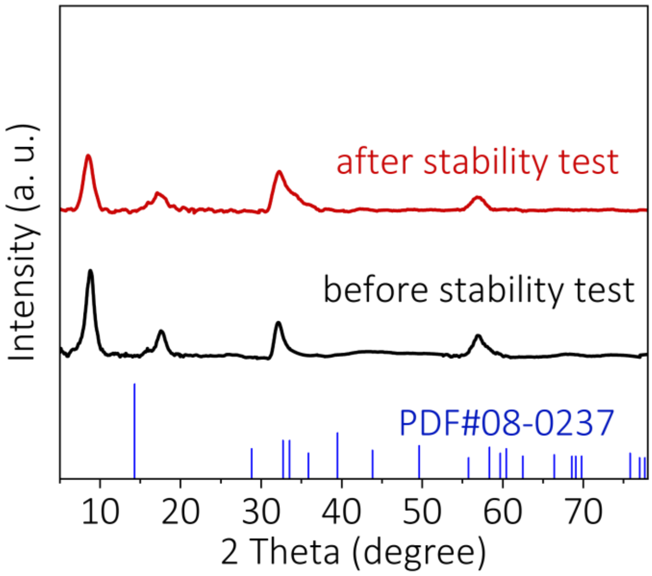

Supplementary Figure 34 | Comparison of XRD patterns for WS_2 superstructure before and after 1000 h AEM electrolyser operation.

XRD patterns reveal that the crystal structure of the used WS_2 superstructure catalyst is the same as that of the pristine catalyst.

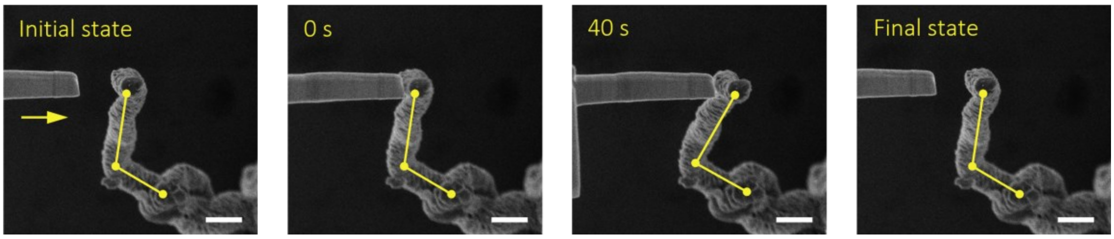

Supplementary Figure 35 | In-situ SEM mechanical test of the WS_2 superstructure post-electrolysis in the AEM electrolyzer. Scale bar, 500 nm.

As shown in Supplementary Fig. 35, the in-situ SEM tests demonstrated the WS_2 superstructure catalyst still maintained excellent mechanical properties after the stability test.

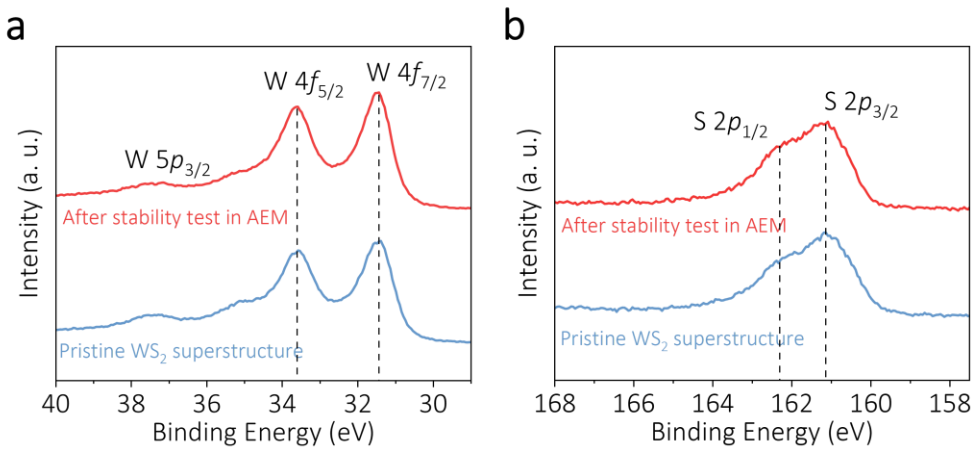

Supplementary Figure 36 | XPS analysis of the WS_2 superstructure anode catalyst before and after AEM elecctrolyzer operation. (a) W 4*f* and (b) S 2*p* XPS signals of the WS_2 superstructure anode catalyst before and after prolonged stability test.

The post-XPS study of the WS_2 superstructure was performed to investigate any possible alteration in the chemical state of the W and S. As for the W 4*f* spectra (Supplementary Fig. 36a), the signals corresponding to the W^{4+} valence states in WS_2 superstructure showed no obvious shifts in binding energies after HER operation in the AEM electrolyser. This result indicated that the W valence state in WS_2 superstructure was relatively stable after AEM electrolyser operation. Similarly, the S 2*p* XPS signals in Supplementary Fig. 36b demonstrated that the S chemical state in WS_2 superstructure was relatively stable after AEM electrolyser operation.

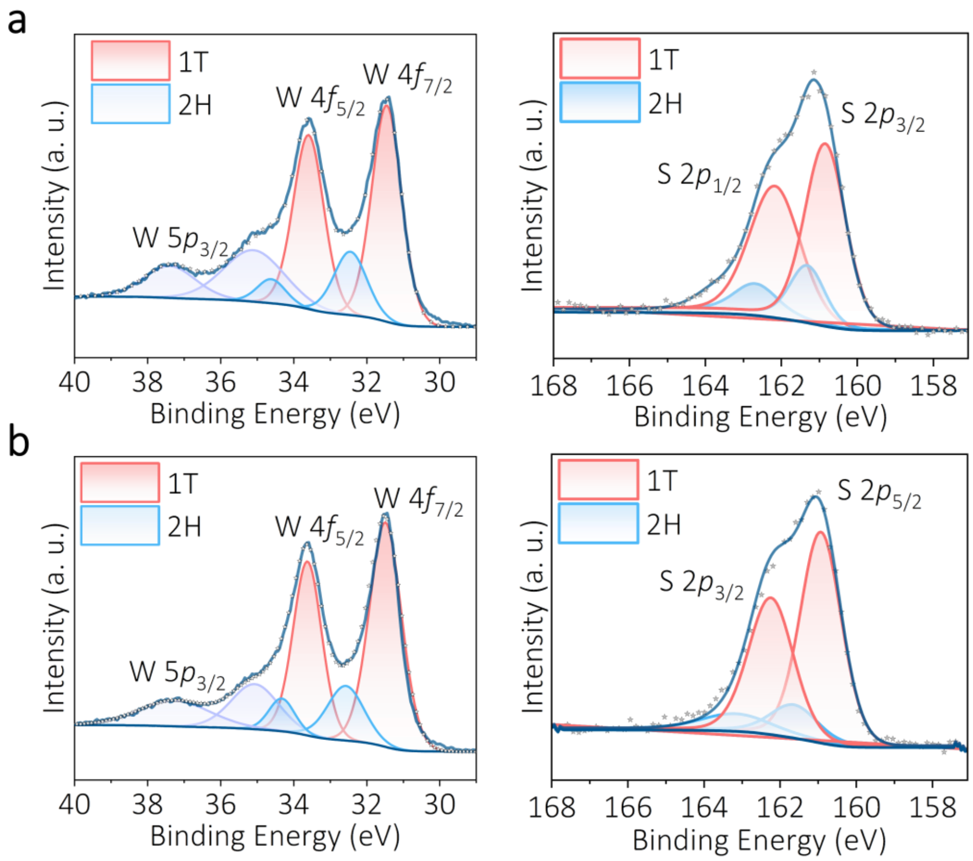

Supplementary Figure 37 | High-resolution XPS spectra of W 4*f* (left) and S 2*p* (right) core level peak regions of the WS_2 superstructure anode catalyst before (a) and after (b) prolonged stability test. The fitting red and blue curves represent the contributions of 1T and 2H phases, respectively.

The tungsten signal is sensitive to its oxidation state and coordination geometry, thus monitoring the position of the binding energy of the W $4f_{7/2}$ and W $4f_{5/2}$ core level peaks allows one to unambiguously distinguish the distinct W species and can be used to determine the relative ratio of 1T and 2H phases in the WS_2 superstructure[3,23].

As shown in Supplementary Fig. 37, double peaks located at 31.8 eV and 33.8 eV are ascribed to the core levels of W $4f_{7/2}$ and W $4f_{5/2}$ of 1T phase WS_2 in the sample,

respectively. Two peaks of WS_2 superstructure at 32.7 eV (W $4f_{7/2}$) and 34.7 eV (W $4f_{5/2}$) are the characteristics of W for 2H phase WS_2[23]. Phase percentages were calculated by peak area ratios of W 4*f* and S 2*p* regions using deconvolution method. As shown in Supplementary Fig. 37a, the relative ratio of 1T phase and 2H phase occupies 82.1% and 17.9% in WS_2 superstructure sample before prolonged stability test, respectively. After the prolonged stability test, the post-XPS revealed that the relative ratio of 1T phase and 2H phase occupies 81.0% and 19.0% in WS_2 superstructure sample (Supplementary Fig. 37b). The similar results were acquired from the research of S 2*p* core level spectra (Supplementary Fig. 37). These results indicated that the chemical state of the W and S in WS_2 superstructure were relatively stable after prolonged stability test.

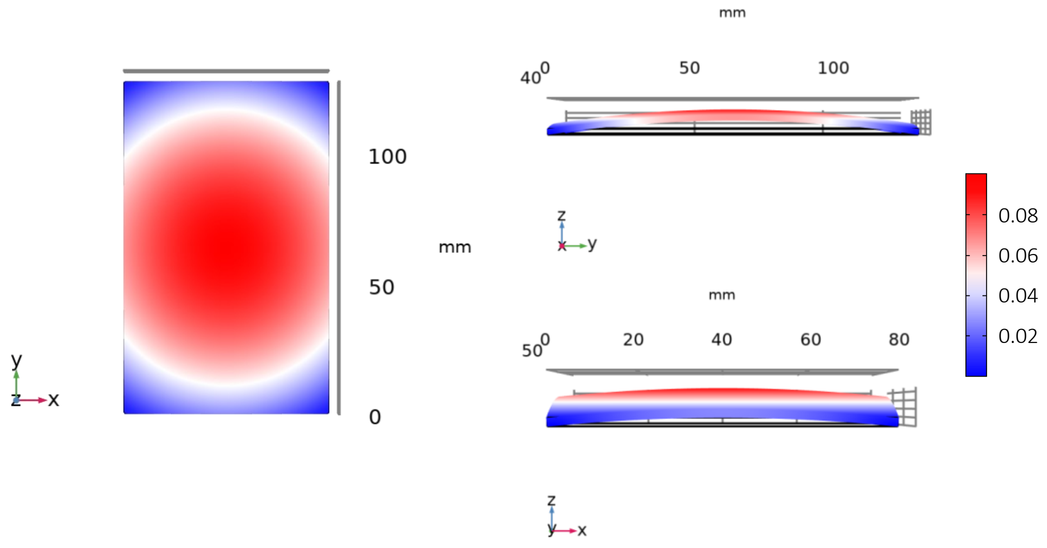

Supplementary Figure 38 | Three views of the thermally induced deformation of the constructed model.

Supplementary Note 1

Electrochemical Active Surface Area (ECSA) Calculations

Cyclic voltammograms (CV) method

Cyclic voltammetry (CV) was conducted to evaluate the electrochemical double layer capacitance (C_{dl}) of the materials at non-faradaic potentials as the means of estimating the corresponding electrochemical active surface areas[24–26]. For CV measurements, a series of CV curves were performed at various scan rates (10, 20, 40, 60, 80, 100, 120, 140, 160, 180 mV s^{-1}) in 0.10–0.30 V vs. RHE region. The cathodic and anodic charging currents measured at 0.20 V vs RHE plotted as a function of scan rate. The determined double-layer capacitance of the system is taken as the average of the absolute value of the slope of the linear fits to the data (Supplementary Figs. 16–19). The specific capacitance values of WS_2 superstructure, 1T-WS_2 and 2H-WS_2 are 19.25 mF cm^{-2}, 14.35 mF cm^{-2}, 0.825 mF cm^{-2} respectively. The background capacitance of the bare CFC electrode was subtracted from the obtained double layer capacitance to compensate for the low substrate coverage (Supplementary Fig. 16).

In general, the ECSA estimates to be accurate within about an order of magnitude, and emphasize that the values should be considered only as an approximate guide for comparing electroactive surface area. The specific capacitance is converted into an electrochemical active surface area (ECSA) using the specific capacitance value for a flat standard with 1 cm^2 of real surface area. The specific capacitance for a flat surface is generally found to be in the range of 20–60 μF cm^{-2}. In the following calculations of ECSA, we assume the value of 60 μF cm^{-2} as the specific capacitance of the catalysts

in this work[27–31]. Based on the above equation analysis, the detailed calculation process is as follows:

Calculated electrochemical active surface area (ECSA):

I: WS_2 superstructure II: 1T-WS_2 III: 2H-WS_2 IV: Pt/C (20 wt%)

$$A^{I}_{ECSA} = \frac{19.25 \text{ mF cm}^{-2}}{60 \text{ μF cm}^{-2} \text{ per cm}^2_{\text{ECSA}}} = 320.8 \text{ cm}^2_{\text{ECSA}}$$

$$A^{II}_{ECSA} = \frac{14.35 \text{ mF cm}^{-2}}{60 \text{ μF cm}^{-2} \text{ per cm}^2_{\text{ECSA}}} = 239.2 \text{ cm}^2_{\text{ECSA}}$$

$$A^{III}_{ECSA} = \frac{0.825 \text{ mF cm}^{-2}}{60 \text{ μF cm}^{-2} \text{ per cm}^2_{\text{ECSA}}} = 13.7 \text{ cm}^2_{\text{ECSA}}$$

$$A^{IV}_{ECSA} = \frac{11.485 \text{ mF cm}^{-2}}{60 \text{ μF cm}^{-2} \text{ per cm}^2_{\text{ECSA}}} = 191.4 \text{ cm}^2_{\text{ECSA}}$$

Supplementary Note 2

Calculation of turn over frequency (TOF)

To calculate the active surface site density and per-site TOF for the WS_2 superstructure catalyst, we adopt the method applied by Jaramillo et al.[32–35], where the relative roughness factor (RF) of the catalyst, the geometry of WS_2 surface, and the HER current density are used. As shown in Supplementary Fig. 19, we have determined the specific capacitance to be 19.25 mF cm^{-2}, which can be directly used to estimate the relevant electrochemical active surface area (ECSA) by using the specific capacitance value for a flat electrode with real surface area 1 cm^2. We assume 60 μF cm^{-2} for a flat electrode provided in Jaramillo et al[32,34] for calculation here.

The number of electrochemically accessible surface sites on the WS_2 superstructure catalyst can be calculated by using the following formula:

$$\frac{\#\,Surface\ sites\ (catalyst)}{cm^2\ geometric\ area} = \frac{\#\,Surface\ sites\ (flat\ standard)}{cm^2\ geometric\ area} \times Roughness\ factor$$

The number of WS_2 units (the number of surface sites for the flat standard) per cm^2 geometric area is 1.164×10^{15}. Compared to the flat standard electrode (60 μF cm^{-2}), the relative roughness factor of the investigated catalyst is determined to be ~320.8 based on the electrochemically double-layer capacitance measurement. As a result, the number of surface active sites for the WS_2 superstructure catalyst is estimated to be 3.7×10^{17} surface sites cm^{-2} from the above formula, indicating a large number of active sites introduced by our special experimental design.

To further get insights into the per-site TOF, the following formula is utilized:

$$TOF\ per\ site = \frac{\#\ Total\ Hydrogen\ Turn\ Over\ /\ cm^2\ geometric\ area}{\#\ Surface\ Sites\ (Catalyst)\ /\ cm^2\ geometric\ area}$$

In this formula, the total number of hydrogen turn over events per geometric area at 1 mA cm^{-2} is close to $3.12 \times 10^{15}\ \frac{H_2/s}{cm^2}$ according to Jaramillo *et al.*'s work[32,36,37]. So the TOF per site for our investigated hybrid catalyst at different overpotentials vs. RHE is calculated as follows:

At η = 100 mV, $\left(3.12 \times 10^{15}\ \frac{H_2/s}{cm^2}/\frac{mA}{cm^2}\right)\left(71.2\ \frac{mA}{cm^2}\right)\left(\frac{1\ cm^2}{3.7 \times 10^{17}\ surface\ sites}\right) = 0.600\ \frac{H_2/s}{surface\ site}$

At η = 200 mV, $\left(3.12 \times 10^{15}\ \frac{H_2/s}{cm^2}/\frac{mA}{cm^2}\right)\left(475.9\ \frac{mA}{cm^2}\right)\left(\frac{1\ cm^2}{3.7 \times 10^{17}\ surface\ sites}\right) = 4.011\ \frac{H_2/s}{surface\ site}$

All the obtained TOF range values are listed in Supplementary Table 3.

Supplementary Note 3

Finite element method simulations

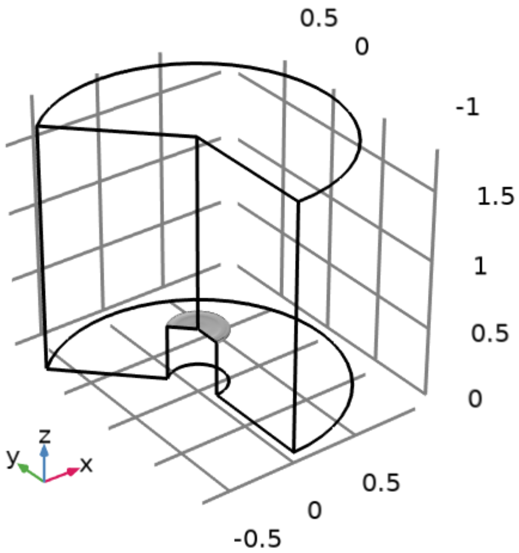

The image above shows the geometry of the model studied in this example. Due to its symmetry, we use an axisymmetric 2D model, which can be further transformed into a 3D structural model.

Representation and convection of the fluid interface

The level set method as well as the phase field method are both well suited for modeling moving boundaries where topology changes occur. The level set interface uses a reinitialized, conservative level set method to describe and convect the fluid interface. The 0.5 contour of the level set function defines the interface, where equals 0 in water and 1 in hydrogen. In a transition layer close to the interface, goes smoothly from 0 to 1. The interface moves with the fluid velocity, u, at the interface. The following equation describes the convection of the reinitialized level set function:

$$\frac{\partial \phi}{\partial t} + \boldsymbol{u} \cdot \nabla \phi + \gamma \left[\left(\nabla \cdot \left(\phi \, (1 - \phi) \frac{\nabla \phi}{|\nabla \phi|} \right) \right) - \varepsilon \nabla \cdot \nabla \phi \right] = 0$$

Here, ϕ as the volume fraction of the hydrogen, $\boldsymbol{u}$ is interface fluid velocity, and γ and ε are reinitialization parameters. The ε parameter determines the thickness of the layer around the interface where goes from zero to one. When stabilization is used for the level set equation, we use an interface thickness of $\varepsilon = h_c/2$, where h_c is the characteristic mesh size in the region passed by the interface. The γ parameter determines the amount of reinitialization. A suitable value for γ is the maximum velocity magnitude occurring in the model.

Mass and momentum transport

In the Laminar Two-Phase Flow, Level Set interface, the transport of mass and momentum is governed by the incompressible Navier-Stokes equations, including surface tension:

$$\rho \left(\frac{\partial \mathbf{u}}{\partial t} + \mathbf{u} \cdot \nabla \mathbf{u} \right) = -\nabla p + \nabla \, \mu(\nabla \mathbf{u} + \nabla \mathbf{u}^T) + \rho g + F_{st}$$

$$\nabla \cdot \mathbf{u} = 0$$

In the above equations, ρ (unit: kg m^{-3}) denotes the density, **u** is the velocity (unit: m s^{-1}), t equals time (unit: s), p is the pressure (unit: Pa), and μ denotes the viscosity (unit: Pa·s). The momentum equations contain gravity, ρg, and surface tension force components, denoted by F_{st}.

Surface Tension

The surface tension force is defined by

$$F_{st} = \nabla\cdot\mathbf{T} = \nabla\cdot[\sigma\{\mathbf{I} + (-\mathbf{n}\mathbf{n}^T)\}\delta]$$

where σ is the surface tension coefficient, **I** is the identity matrix, **n** is the interface unit normal, and δ is a Dirac delta function, nonzero only at the fluid interface. The interface normal is calculated from

$$n = \frac{\nabla\phi}{|\nabla\phi|}$$

while the delta function is approximated by

$$\delta = 6|\phi(1-\phi)||\nabla\phi|$$

For the model in this work, use no slip conditions, **u** = 0 at the top and bottom and a wetted wall condition on the right boundary. The left boundary corresponds to the symmetry axis. As outlined above, the topology of the fluid interface changes with time.

Supplementary Note 3

Numerical simulations of the fluid-structure interaction

The model geometry consists of a horizontal flow channel in the middle of which is an obstacle, the narrow vertical structure with different material characteristics. The fluid flows from left to right, except where the obstacle forces it into a narrow path in the upper part of the channel, and it imposes a force on the structure's walls resulting from the viscous drag and fluid pressure. The structure, being made of a deformable material (with different Young's modulus), may bends under the applied load.

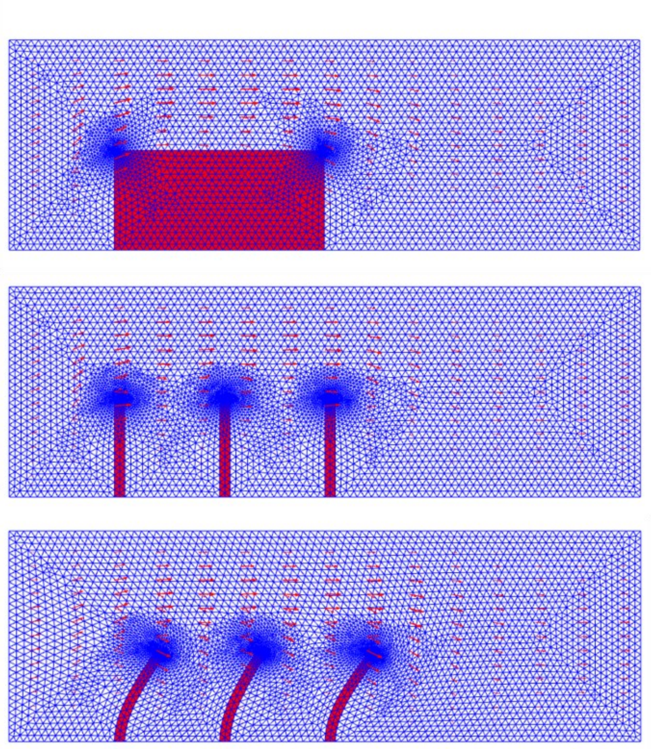

Fluid Flow

The fluid flow in the channel is described by the incompressible Navier-Stokes equations

for the velocity field, $\mathbf{u} = (u, v)$, and the pressure, p, in the spatial (deformed) moving coordinate system:

$$\rho \frac{\partial \mathbf{u}}{\partial t} - \nabla[-p\mathbf{I} + \eta(\nabla\mathbf{u} + \nabla\mathbf{u}^T)] + \rho\big((\mathbf{u} - \mathbf{u}_m)\nabla\big)\mathbf{u} = \mathbf{F}$$

$$-\nabla\cdot\mathbf{u} = 0$$

In these equations, **I** denotes the unit diagonal matrix and **F** is the volume force affecting the fluid. Assume that no gravitation or other volume forces affect the fluid, so that **F** = 0. The coordinate system velocity is $\mathbf{u}_m = (u_m, v_m)$.

Structural Mechanics

The structural deformations are solved for using an elastic formulation and a nonlinear geometry formulation to allow large deformations. The obstacle is fixed to the bottom of the fluid channel. All other object boundaries experience a load from the fluid, given by

$$-\mathbf{n}[-p\mathbf{I} + \eta(\nabla\mathbf{u} + \nabla\mathbf{u}^T)] = \mathbf{F_T}$$

where **n** is the normal vector to the boundary. This load represents a sum of pressure and viscous forces.

Moving Mesh

The Navier-Stokes equations are solved on a freely moving deformed mesh, which constitutes the fluid domain. The deformation of this mesh relative to the initial shape

of the domain is computed using Yeoh smoothing. Inside the obstacle, the moving mesh follows the deformations of the obstacle. At the exterior boundaries of the flow domain, the deformation is zero in all directions.

Supplementary Note 4

DFT calculation

All the spin-polarized DFT calculations were performed by the Vienna *ab initio* simulation package (VASP) code with the method of projector augmented wave (PAW)[38]. The generalized gradient approximation (GGA) with Perdew-Burke-Ernzerhof (PBE) functional was utilized to describe the exchange-correlation potential[39]. The Grimme method (DFT-D3 correction) was adopted to accurately describe the van der Waals interactions[40]. A 15 Å vacuum space was used along the z direction i.e. perpendicular to the 2D sheet, to avoid spurious interaction among the periodic images. An energy cut-off of 500 eV for the plane-wave expansion and Monkhorst-Pack k-point meshes of 7 × 7 × 1 in the 2D Brillouin zone (BZ) were used for the geometry optimization of new W_2S_3 structural calculation. During the geometry optimization, the atomic position and lattice vectors were fully relaxed until energy and force were converged to 10^{-4} eV and 0.01 eV Å^{-1}, respectively. The phonon dispersion analysis was carried out for the low-lying allotropes using the Phonopy code with the finite displacement method, interfaced with density functional perturbation theory implemented in VASP[41]. To obtain reliable results of phonon dispersion, more accurate DFT calculations were performed for further geometry optimization accuracy. The more stringent energy convergence criterion was set to 10^{-8} eV for the total energy and 10^{-4} eV Å^{-1} for the force convergence during the phonon calculations. The Gibbs free energy changes were evaluated according to $\Delta G = \Delta E_{H} + \Delta E_{ZPE} - T\Delta S$, where ΔE_{H} is the hydrogen adsorption energy, ΔE_{ZPE} and ΔS are the zero point energy difference and

the entropy difference between the adsorbed state and the gas phase, respectively, and T is the system temperature (298.15 K). The climbing image nudged elastic band (CI-NEB) method was used to find saddle points and minimum energy paths, and the transition state was confirmed by frequency analysis where there is only one imaginary frequency in a transition state[42].

Supplementary Table 1 | Comparison of the HER performance of Pt/C sample in this work with some previously reported Pt/C data in alkaline media.

**Catalyst**	**Electrolyte**	**η@1000 mA cm^{-2} (mV)**	**Reference**
Pt/C-NF	1 M KOH	490	43
Pt/C-NF	1 M KOH	780	44
Pt/C	1 M KOH	410	45
Pt/C-NF	1 M KOH	420	46
Pt/C-Cu	1 M KOH	450	47
Pt/C-NF	1 M KOH	462	48
Pt/C-NF	1 M KOH	650 @400 mA cm^{-2}	49
Pt/C-NF	1 M KOH	370 @500 mA cm^{-2}	50
Pt/C-NF	1 M KOH	350 @460 mA cm^{-2}	51
Pt/C	1 M KOH	520 @600 mA cm^{-2}	52
Pt/C	1 M KOH	240 @400 mA cm^{-2}	53
Pt/C	1 M KOH	230 @800 mA cm^{-2}	54
**Pt/C**	**1 M KOH**	**~430**	**This work**

Supplementary Table 2 | The ECSA, TOF, and ECSA-normalized current density values of different catalysts.

Catalyst	ECSA (cm^2)	TOF (s^{-1}) @overpotential (mV)	J_{ECSA} (η = −300 mV vs. RHE) (mA cm^{-2})
WS_2 superstructure	320.8	0.600@100<br>4.011@200	3.53
Pt/C (20 wt%)	191.4	0.940@100<br>2.670@200	2.61

Supplementary Table 3 | The TOF values of various WS_2 based catalysts.

Catalyst	Potential (mV)	TOF (s^{-1})	Active site density (cm^{-2})
WS_2 superstructure	100	0.600	3.7×10^{17}
	200	4.011	
	300	11.235	
1T-WS_2 nanosheets	100	0.116	2.7×10^{17}
	200	0.697	
	300	1.592	
2H-WS_2 nanosheets	100	0.419	1.6×10^{16}
	200	1.07835	
	300	1.143	

Supplementary Table 4 | Comparison of TOF values of HER catalysts in alkaline condition.

Catalyst	TOF (H_2 s^{-1}@mV)	Reference
$NiCo_2P_x$	0.056 s^{-1} at 100 mV	55
$MoNi_4/MoO_{3-x}$	1.13 s^{-1} at 100 mV	56
N-$NiCo_2S_4$	1.0 s^{-1} at 125 mV	57
NiO@1T MoS_2	0.7 s^{-1} at 130 mV	58
$Mo_1N_1C_2$	1.46 s^{-1} at 150 mV	59
Co-NiS_2	4.1 s^{-1} at 200 mV	60
MoO_3@MoS_2	1.93 s^{-1} at 250 mV	61
$NiMoO_x$/NiMoS	0.28 s^{-1} at 50 mV	62
$NiMoO_x$/NiMoS	1.97 s^{-1} at 100 mV	62
**WS_2 superstructure**	**0.600 s^{-1} at 100 mV**	**This work**
**WS_2 superstructure**	**4.011 s^{-1} at 200 mV**	**This work**

Supplementary Table 5 | Comparison of the HER activity of WS_2 superstructure and some representative high current density HER electrocatalysts.

Catalyst	Electrolyte	η@1000 mA/cm^2 (mV)	Reference
Ni_2P/NF	1 M KOH	306	43
α-MoB_2	1 M KOH	334	44
MoS_2/Mo_2C	1 M KOH	440	45
MoS_2/Ni_3S_2	1 M KOH	200	46
$CuMo_6S_8$/Cu	1 M KOH	320	47
S-NiBDC	1 M KOH	310	48
MoS_2/Mo_2C	1 M KOH	224	63
FeP/Ni_2P	1 M KOH	~265	64
$NiMoO_x$/NiMoS	1 M KOH	236	65
Mo_2C/MoC/CNT	1 M KOH	233	66
2H-$Nb_{1+x}S_2$	0.5 M H_2SO_4	~370	67
$Ni_{2(1-x)}Mo_{2x}P$	1 M KOH	294	68
Ni_2P-Fe_2P	1 M KOH	389	69
Sn–Ni_3S_2	1 M KOH	570	70

LiCoBPO/NF	1 M KOH	400	71
NiCo LDH/NF	1 M KOH	381	72
Co/Se-MoS_2	0.5 M H_2SO_4	382	73
Ta/TaS_2 MC	0.5 M H_2SO_4	295	74
IrFe	1 M KOH	850	75
Sr_2RuO_4	1 M KOH	278	76
	0.5 M H_2SO_4	182	76
Ni_3S_2/Cr_2S_3	1 M KOH	227	77
Ru clusters	1 M KOH	196	78
Cu-FeOOH/Fe_3O_4	1 M KOH	349	79
IrNi-$FeNi_3$	1 M KOH	~289	80
Ru-CoO_x	1 M KOH	252	81
P-NiMoHZ	1 M KOH	210	82
NiP_2-FeP_2/Cu	1 M KOH	357	83
C-$Ni_{1-x}O$	1 M KOH	245	84
CoOx/RuO_2	1 M KOH	200	85
**WS_2 superstructure**	**1 M KOH**	**264**	**This work**

Supplementary Table 6 | Impedance parameters for the equivalent circuit that was shown in **Fig. 4d**.

Samples	R_s	CPE-T	CPE-P	R_{ct}
WS_2 Superstructure	0.486	1.08×10^{-3}	0.77	3.87
1T-WS_2 nanosheets	0.562	4.01×10^{-4}	0.80	8.16
2H-WS_2 nanosheets	0.697	5.33×10^{-4}	0.76	17.14

Supplementary Table 7 | Comparison of the AEM activity for WS_2 superstructure electrocatalyst with other reported electrocatalysts.

Catalyst	Cell voltage (V)	Degradation rate (μV/h)	Reference
Fe-NiMo-NH_3/H_2‖NiMo-NH_3/H_2	1.77@1 A cm^{-2}	~1600	86
NA-Ru_3Ni/C‖NA-Ru_3Ni/C	2.05@1 A cm^{-2}	54	87
Pt/C‖IrO_2/C	2.49@1 A cm^{-2}	4482	87
Ni_3S_2/Cr_2S_3@NF‖NiFeCr-LDH@NF	2.04@1 A cm^{-2}	4021	88
FeP–CoP/NC‖FeP–CoP/NC	2.10@0.75 A cm^{-2}	1575	89
LSC&$MoSe_2$‖LSC&$MoSe_2$	2.30@1 A cm^{-2}	221	90
VCoP-2/Ni‖VCoP-2/Ni	~1.90@1 A cm^{-2}	2500	91
NFN-MOF/NF	~1.85@0.25 A cm^{-2}	3013	92
Acta 3030‖Acta 4030	1.90@0.5 A cm^{-2}	810	93
NiFe‖PtRu/C	1.80@2.7 A cm^{-2}	1170	94
IrO_2‖Pt black	~1.75@0.5 A cm^{-2}	670	95
MOC-Ru‖RuO_2	1.97@0.25 A cm^{-2}	409	96

C-Ru||RuO_2	2.03@0.25 A cm^{-2}	3227	
NiCoP@NiFeP||NiCoP@NiFeP	1.93@1 A cm^{-2}	500	97
Co, Mo-NiFe LDH||Pt/C	1.94@2 A cm^{-2}	154	98
**IrO_2||WS_2 superstructure**	**1.70@1 A cm^{-2}**	**9.67**	**This work**

Supplementary References

1. Shi, Z.-T., Kang, W., Xu, J., Sun, Y.-W., Jiang, M., Ng, T.-W., Xue, H.-T., Yu, D.Y.W., Zhang, W., Lee, C.-S. Hierarchical nanotubes assembled from MoS_2-carbon monolayer sandwiched superstructure nanosheets for high-performance sodium ion batteries. *Nano Energy* **22**, 27-37 (2016).
2. Du, Y., Yin, Z., Zhu, J., Huang, X., Wu, X.J., Zeng, Z., Yan, Q., Zhang, H. A general method for the large-scale synthesis of uniform ultrathin metal sulphide nanocrystals. *Nat. Commun.* **3**, 1177 (2012).
3. Liu, Z., Li, N., Su, C., Zhao, H., Xu, L., Yin, Z., Li, J., Du, Y. Colloidal synthesis of 1T' phase dominated WS_2 towards endurable electrocatalysis. *Nano Energy* **50**, 176-181 (2018).
4. Xie, L., Wang, L., Liu, X., Zhao, W., Liu, S., Huang, X., Zhao, Q. Tetra-coordinated W_2S_3 for efficient dual-pH hydrogen production. *Angew. Chem. Int. Ed.* **63**, e202316306 (2024).
5. Willcox, J.A., Kim, H.J. Molecular dynamics study of water flow across multiple layers of pristine, oxidized, and mixed regions of graphene Ooxide. *ACS Nano* **11**, 2187-2193 (2017).
6. Xu, P., von Rueden, A.D., Schimmenti, R., Mavrikakis, M., Suntivich, J. Optical method for quantifying the potential of zero charge at the platinum-water electrochemical interface. *Nat. Mater.* **22**, 503-510 (2023).
7. Chen, W. et al. Two-dimensional quantum-sheet films with sub-1.2 nm channels for ultrahigh-rate electrochemical capacitance. *Nat. Nanotechnol.* **17**, 153-158

(2022).

8. Wang, Y.-H., Zheng, S., Yang, W.-M., Zhou, R.-Y., He, Q.-F., Radjenovic, P., Dong, J.-C., Li, S., Zheng, J., Yang, Z.-L., Attard, G., Pan, F., Tian, Z.-Q., Li, J.-F. In situ Raman spectroscopy reveals the structure and dissociation of interfacial water. *Nature* **600**, 81-85 (2021).
9. Li, C.Y., Le, J.B., Wang, Y.H., Chen, S., Yang, Z.L., Li, J.F., Cheng, J., Tian, Z.Q. In situ probing electrified interfacial water structures at atomically flat surfaces. *Nat. Mater.* **18**, 697-701 (2019).
10. Wang, Y.-H., Li, S., Zhou, R.-Y., Zheng, S., Zhang, Y.-J., Dong, J.-C., Yang, Z.-L., Pan, F., Tian, Z.-Q., Li, J.-F. In situ electrochemical Raman spectroscopy and ab initio molecular dynamics study of interfacial water on a single-crystal surface. *Nat. Protoc.* **18**, 883-901 (2023).
11. Wang, J., Yang, T., Li, X., Zhang, H., Zhang, Y., He, Y., Xue, H. Hydrogen evolution reaction activity enhancement from active site turnover mechanism. *J. Energy Chem.* **92**, 629-638 (2024).
12. Yang, C., Yue, J., Wang, G., Luo, W. Activating and identifying the active site of RuS_2 for alkaline hydrogen oxidation electrocatalysis. *Angew. Chem. Int. Ed.*, e202401453 (2024).
13. Chen, J., Liu, G., Zhu, Y.-z., Su, M., Yin, P., Wu, X.-j., Lu, Q., Tan, C., Zhao, M., Liu, Z., Yang, W., Li, H., Nam, G.-H., Zhang, L., Chen, Z., Huang, X., Radjenovic, P.M., Huang, W., Tian, Z.-q., Li, J.-f., Zhang, H. Ag@MoS_2 core–shell heterostructure as SERS platform to reveal the hydrogen evolution active sites of

single-layer MoS_2. *J. Am. Chem. Soc.* 142, 7161-7167 (2020).

14. Guo, S., Li, Y., Tang, S., Zhang, Y., Li, X., Sobrido, A.J., Titirici, M.M., Wei, B. Monitoring hydrogen evolution reaction intermediates of transition metal dichalcogenides via operando Raman spectroscopy. *Adv. Funct. Mater.* **30**, 2003035 (2020).
15. Wang, X., Andrews, L. Neon matrix infrared spectra and DFT calculations of tungsten hydrides WH_x (x = 1−4, 6). *J. Phys. Chem. A* **106**, 6720-6729 (2002).
16. Deng, Y., Ting, L.R.L., Neo, P.H.L., Zhang, Y.-J., Peterson, A.A., Yeo, B.S. Operando Raman spectroscopy of amorphous molybdenum sulfide (MoS_x) during the electrochemical hydrogen evolution reaction: Identification of sulfur atoms as catalytically active sites for H^+ reduction. *ACS Catal.* **6**, 7790-7798 (2016).
17. Russell, A.E. Hydrogen evolution on nano-particulate transition metal sulfides. *Faraday Discuss.* **140**, 9-10 (2008).
18. Miao, J., Xiao, F.-X., Yang, H.B., Khoo, S.Y., Chen, J., Fan, Z., Hsu, Y.-Y., Chen, H.M., Zhang, H., Liu, B. Hierarchical Ni-Mo-S nanosheets on carbon fiber cloth: A flexible electrode for efficient hydrogen generation in neutral electrolyte. *Sci. adv.* **1**, e1500259 (2015).
19. Hu, C., Ma, Q., Hung, S.-F., Chen, Z.-N., Ou, D., Ren, B., Chen, H.M., Fu, G., Zheng, N. In situ electrochemical production of ultrathin nickel nanosheets for hydrogen evolution electrocatalysis. *Chem* **3**, 122-133 (2017).
20. Tang, C., Wang, H.F., Zhang, Q. Multiscale principles to boost reactivity in gas-involving energy electrocatalysis. *Acc. Chem. Res.* **51**, 881-889 (2018).

21. Wang, J., Liang, C., Ma, X., Liu, P., Pan, W., Zhu, H., Guo, Z., Sui, Y., Liu, H., Liu, L., Yang, C. Dynamically adaptive bubbling for upgrading oxygen evolution reaction using lamellar fern-Like alloy aerogel self-standing electrodes. *Adv. Mater.* **36**, e2307925 (2024).

22. Ren, G., Zhou, M., Hu, P., Chen, J.F., Wang, H. Bubble-water/catalyst triphase interface microenvironment accelerates photocatalytic OER via optimizing semi-hydrophobic OH radical. *Nat. Commun.* **15**, 2346 (2024).

23. Xie, L., Wang, L., Zhao, W., Liu, S., Huang, W., Zhao, Q. WS_2 moiré superlattices derived from mechanical flexibility for hydrogen evolution reaction. *Nat. Commun.* **12**, 5070 (2021).

24. Kibsgaard J., Jaramillo TF., Besenbacher F. Building an appropriate active-site motif into a hydrogen-evolution catalyst with thiomolybdate $[Mo_3S_{13}]^{2-}$ clusters. *Nat. Chem.* **6**, 248–253 (2014).

25. McCrory CC., Jung S., Peters JC., Jaramillo TF. Benchmarking heterogeneous electrocatalysts for the oxygen evolution reaction. *J. Am. Chem. Soc.* **135**, 16977–16987 (2013).

26. Kibsgaard J., Jaramillo TF. Molybdenum phosphosulfide: An active, acid-stable, earth-abundant catalyst for the hydrogen evolution reaction. *Angew. Chem. Int. Ed.* **53**, 14433–14437 (2014).

27. McCrory CC., Jung S., Ferrer IM., Chatman SM., Peters JC., Jaramillo TF. Benchmarking hydrogen evolving reaction and oxygen evolving reaction

electrocatalysts for solar water splitting devices. *J. Am. Chem. Soc.* **137**, 4347–4357 (2015).

28. Kibsgaard J., et al. Designing an improved transition metal phosphide catalyst for hydrogen evolution using experimental and theoretical trends. *Energy Environ. Sci.* **8**, 3022–3029 (2015).
29. Gauthier JA., et al. Transition metal arsenide catalysts for the hydrogen evolution reaction. *J. Phys. Chem. C* **123**, 24007–24012 (2019).
30. Benck JD., Hellstern TR., Kibsgaard J., Chakthranont P., Jaramillo TF. Catalyzing the hydrogen evolution reaction (HER) with molybdenum sulfide nanomaterials. *ACS Catal.* **4**, 3957–3971 (2014).
31. Li H., et al. Amorphous nickel-cobalt complexes hybridized with 1T-phase molybdenum disulfide via hydrazine-induced phase transformation for water splitting. *Nat. Commun.* **8**, 15377 (2017).
32. Benck JD., Chen Z., Kuritzky LY., Forman AJ., Jaramillo TF. Amorphous molybdenum sulfide catalysts for electrochemical hydrogen production: insights into the origin of their catalytic activity. *ACS Catal.* **2,** 1916–1923 (2012).
33. Sanchez J., Hellstern TR., King LA., Jaramillo TF. Surface engineering of 3D gas diffusion electrodes for high-performance H_2 production with nonprecious metal catalysts. *Adv. Energy Mater.* **9**, 1901824 (2019).
34. King LA., et al. A non-precious metal hydrogen catalyst in a commercial polymer electrolyte membrane electrolyser. *Nat. Nanotechnol.* **14**, 1071-1074 (2019).

35. Kibsgaard J., Chen Z., Reinecke BN., Jaramillo TF. Engineering the surface structure of MoS_2 to preferentially expose active edge sites for electrocatalysis. *Nat. Mater.* **11**, 963–969 (2012).

36. Hellstern TR., Benck JD., Kibsgaard J., Hahn C., Jaramillo TF. Engineering cobalt phosphide (CoP) thin film catalysts for enhanced hydrogen evolution activity on silicon photocathodes. *Adv. Energy Mater.* **6**, 1501758 (2016).

37. Hellstern TR., et al. Investigating catalyst–support interactions to improve the hydrogen evolution reaction activity of thiomolybdate $[Mo_3S_{13}]^{2-}$ nanoclusters. *ACS Catal.* **7**, 7126–7130 (2017).

38. Kresse, G., Furthmüller, J. Efficient iterative schemes for ab initio total-energy calculations using a plane-wave basis set. *Phys. Rev. B* **54**, 11169-11186 (1996).

39. Perdew, J.P., Burke, K., Ernzerhof, M. Generalized gradient approximation made simple. *Phys. Rev. Lett.* **77**, 3865-3868 (1996).

40. Burns, L.A., Mayagoitia, Á.V.-., Sumpter, B.G., Sherrill, C.D. Density-functional approaches to noncovalent interactions: A comparison of dispersion corrections (DFT-D), exchange-hole dipole moment (XDM) theory, and specialized functionals. *J. Chem. Phys.* **134**, 084107 (2011).

41. Togo, A., Tanaka, I. First principles phonon calculations in materials science. *Scr. Mater.* **108**, 1-5 (2015).

42. Henkelman, G., Uberuaga, B.P., Jónsson, H. A climbing image nudged elastic band method for finding saddle points and minimum energy paths. *J. Chem. Phys.* **113**, 9901-9904 (2000).

43. Yu, X. et. al. “Superaerophobic” nickel phosphide nanoarray catalyst for efficient hydrogen evolution at ultrahigh current densities. *J. Am. Chem. Soc.* **141**, 7537 (2019).

44. Chen Y, et al. Highly active nonprecious electrocatalyst comprising borophene subunits for the hydrogen evolution reaction. *J. Am. Chem. Soc.* **139**, 12370-12373 (2017).

45. Zhang C, et al. High-throughput production of cheap mineral-based two-dimensional electrocatalysts for high-current-density hydrogen evolution. *Nat Commun.* **11**, 3724 (2020).

46. Xue, S., Liu, Z., Ma, C., Cheng, H. M., Ren, W. A highly active and durable electrocatalyst for large current density hydrogen evolution reaction. *Sci. Bull.* **65**, 123-130 (2020).

47. Liu H., et al. Dual interfacial engineering of a Chevrel phase electrode material for stable hydrogen evolution at 2500 mA cm^{-2}. *Nat. Commun.* **13**, 6382 (2022).

48. Cheng, F. et al. Accelerated water activation and stabilized metal-organic framework via constructing triangular active-regions for ampere-level current density hydrogen production. *Nat. Commun.* **13**, 6486 (2022).

49. Jian, J. et al. Metal-ionic-conductor potassium ferrite nanocrystals with intrinsic superhydrophilic surfaces for electrocatalytic water splitting at ultrahigh current densities. *J. Mater. Chem. A* **9**, 7586-7593 (2021).

50. Yu, M., Wang, Z., Liu, J., Sun, F., Yang, P., Qiu, J. A hierarchically porous and hydrophilic 3D nickel-iron/MXene electrode for accelerating oxygen and hydrogen evolution at high current densities. *Nano Energy* **63**, 103880 (2019).

51. Raja, D. S., Lin, H. W., Lu, S. Y. Synergistically well-mixed MOFs grown on nickel foam as highly efficient durable bifunctional electrocatalysts for overall water splitting at high current densities. *Nano Energy* **57**, 1-13 (2019).

52. Shan, X. et al. An engineered superhydrophilic/superaerophobic electrocatalyst composed of the supported $CoMoS_x$ chalcogel for overall water splitting. *Angew. Chem. Int. Ed.* **59**, 1659-1665 (2020).

53. Jiang, N., You, B., Sheng, M., Sun, Y. Electrodeposited cobalt-phosphorous-derived films as competent bifunctional catalysts for overall water splitting. *Angew. Chem. Int. Ed.* **127**, 6349-6352 (2015).

54. Yu, C. et al. Bimetallic Ni-Co phosphide nanosheets self-supported on nickel foam as high-performance electrocatalyst for hydrogen evolution reaction. *Electrochim. Acta* **317**, 191-198 (2019).

55. Zhang, R. et al. Ternary $NiCo_2P_x$ nanowires as pH-universal electrocatalysts for highly efficient hydrogen evolution reaction. *Adv. Mater.* **29**, 1605502 (2017).

56. Chen, Y. et al. Self-templated fabrication of $MoNi_4/MoO_{3-x}$ nanorod arrays with dual active components for highly efficient hydrogen evolution. *Adv. Mater.* **29**, 1703311 (2017).

57. Wu, Y. et al. Electron density modulation of $NiCo_2S_4$ nanowires by nitrogen incorporation for highly efficient hydrogen evolution catalysis. *Nat. Commun.* **9**, 1425 (2018).

58. Huang, Y. et al. Atomically engineering activation sites onto metallic 1T-MoS_2 catalysts for enhanced electrochemical hydrogen evolution. *Nat. Commun.* **10**, 982 (2019).

59. Chen, W. et al. Rational design of single molybdenum atoms anchored on n-doped carbon for effective hydrogen evolution reaction. *Angew. Chem. Int. Ed.* **56**, 16086-16090 (2017).

60. Yin, J. et al. Atomic arrangement in metal-doped NiS_2 boosts the hydrogen evolution reaction in alkaline media. *Angew. Chem. Int. Ed.* **58**, 18676-18682 (2019).

61. Huang, L. et al. Self-limited on-site conversion of MoO_3 nanodots into vertically aligned ultrasmall monolayer MoS_2 for efficient hydrogen evolution. *Adv. Energy Mater.* **8**, 1800734 (2018).

62. Zhai P., et al. Engineering active sites on hierarchical transition bimetal oxides/sulfides heterostructure array enabling robust overall water splitting. *Nat. Commun.* **11**, 5462 (2020).

63. Luo, Y. et al. Morphology and surface chemistry engineering toward pH-universal catalysts for hydrogen evolution at high current density. *Nat. Commun.* **10**, 269 (2019).

64. Yu, F. et. al. High-performance bifunctional porous non-noble metal phosphide catalyst for overall water splitting. *Nat. Commun.* **9**, 2551 (2018).

65. Zhai, P. et al. Engineering active sites on hierarchical transition bimetal oxides/sulfides heterostructure array enabling robust overall water splitting. *Nat. Commun.* **11**, 5462 (2020).

66. Li, C. et al. Ultrafast self-heating synthesis of robust heterogeneous nanocarbides for high current density hydrogen evolution reaction. *Nat. Commun.* **13**, 3338 (2022).

67. Yang, J. et al. Ultrahigh-current-density niobium disulfide catalysts for hydrogen evolution. *Nat. Mater.* **18**, 1309-1314 (2019).

68. Yu, L. et al. Ternary $Ni_{2(1-x)}Mo_{2x}P$ nanowire arrays toward efficient and stable hydrogen evolution electrocatalysis under large-current-density. *Nano Energy* **53**, 492-500 (2018).

69. Wu, L. et al. Heterogeneous bimetallic phosphide Ni_2P-Fe_2P as an efficient bifunctional catalyst for water/seawater splitting. *Adv. Funct. Mater.* **31**, 2006484 (2020).

70. Jian, J. et al. Sn-Ni_3S_2 ultrathin nanosheets as efficient bifunctional water-splitting catalysts with a large current density and low overpotential. *ACS Appl. Mater. Interfaces* **10**, 40568-40576 (2018).

71. Menezes PW, et al. Helical cobalt borophosphates to master durable overall water-splitting. *Energy Environ. Sci.* **12**, 988-999 (2019).

72. Yang, H. Chen Z, Guo P, Fei B, Wu R. B-doping-induced amorphization of LDH for large-current-density hydrogen evolution reaction. *Appl. Catal. B: Environ.* **261**, 118240 (2020).

73. Zheng, Z. et al. Boosting hydrogen evolution on MoS_2 via co-confining selenium in surface and cobalt in inner layer. *Nat. Commun.* **11**, 3315 (2020).

74. Yu, Q. Zhang Z, Qiu S. A Ta-TaS_2 monolith catalyst with robust and metallic interface for superior hydrogen evolution. *Nat. Commun.* **12**, 6051 (2021).

75. Jiang, P. et al. Improving electrocatalytic activity of iridium for hydrogen evolution at high current densities above 1000 mA cm^{-2}. *Appl. Catal. B: Environ.* **258**, 117965 (2019).

76. Zhang, Y. et al. Observation of a robust and active catalyst for hydrogen evolution under high current densities. *Nat. Commun.* **13**, 7784 (2022).

77. Fu, H. et al. Hydrogen spillover-bridged volmer/tafel processes enabling ampere-level current density alkaline hydrogen evolution reaction under low overpotential. *J. Am. Chem. Soc.* **144**, 6028-6039 (2022).

78. Hu, Q. et al. Subnanometric Ru clusters with upshifted D band center improve performance for alkaline hydrogen evolution reaction. *Nat. Commun.* **13**, 3958 (2022).

79. Yang, C. et al. Electrochemically reconstructed Cu-FeOOH/Fe_3O_4 catalyst for efficient hydrogen evolution in alkaline media. *Adv. Energy Mater.*, 2200077 (2022).

80. Wang, Y. et al. Industrially promising IrNi-$FeNi_3$ hybrid nanosheets for overall water splitting catalysis at large current density. *Appl. Catal. B: Environ.* **286**, 119881 (2021).

81. Wu, D., Chen, D., Zhu, J., Mu, S. Ultralow Ru incorporated amorphous cobalt-based oxides for high-current-density overall water splitting in alkaline and seawater media. *Small* **17**, 2102777 (2021).

82. Wang, Z. et al. Manipulation on active electronic states of metastable phase beta-$NiMoO_4$ for large current density hydrogen evolution. *Nat. Commun.* **12**, 5960 (2021).

83. Kumar, A. et al. Modulating interfacial charge density of NiP_2–FeP_2 via coupling with metallic Cu for accelerating alkaline hydrogen evolution. *ACS Energy Lett.* **6**, 354-363 (2021).

84. Kou, T. et al. Periodic porous 3D electrodes mitigate gas bubble traffic during alkaline water electrolysis at high current densities. *Adv. Energy Mater.* **10**, 2002955 (2020).

85. Yu, T. et al. Amorphous CoO_x-decorated crystalline RuO_2 nanosheets as bifunctional catalysts for boosting overall water splitting at large current density. *ACS Sustainable Chem. Eng.* **8**, 17520-17526 (2020).

86. Chen, P., Hu, X. High-efficiency anion exchange membrane water electrolysis employing non-noble metal catalysts. *Adv. Energy Mater.* **10**, 2002285 (2020).

87. Gao, L. et al. Engineering a local potassium cation concentrated microenvironment toward the ampere-level current density hydrogen evolution reaction. *Energy Environ. Sci.* **16**, 285-294 (2023).

88. Fu, H.Q. et al. Hydrogen spillover-bridged volmer/tafel processes enabling ampere-level current density alkaline hydrogen evolution reaction under low overpotential. *J. Am. Chem. Soc.* **144**, 6028-6039 (2022).

89. Yan, X. et al. A membrane-free flow electrolyzer operating at high current density using earth-abundant catalysts for water splitting. *Nat. Commun.* **12**, 4143 (2021).

90. Oh, N.K. et al. In-situ local phase-transitioned $MoSe_2$ in $La_{0.5}Sr_{0.5}CoO_{3-\delta}$ heterostructure and stable overall water electrolysis over 1000 hours. *Nat. Commun.* **10**, 1723 (2019).

91. Wan, L., Xu, Z., Xu, Q., Wang, P., Wang, B. Overall design of novel 3D-ordered MEA with drastically enhanced mass transport for alkaline electrolyzers. *Energy Environ. Sci.* **15**, 1882-1892 (2022).

92. Senthil Raja, D., Chuah, X.-F., Lu, S.-Y. In situ grown bimetallic MOF-based composite as highly efficient bifunctional electrocatalyst for overall water splitting with ultrastability at high current densities. *Adv. Energy Mater.* **8**, 1801065 (2018).

93. Razmjooei, F. et al. Increasing the performance of an anion-exchange membrane electrolyzer operating in pure water with a nickel-based microporous layer. *Joule* **5**, 1776-1799 (2021).

94. Li, D. et al. Highly quaternized polystyrene ionomers for high performance anion exchange membrane water electrolysers. *Nat. Energy* **5**, 378-385 (2020).

95. Lindquist, G.A. et al. Performance and durability of pure-water-fed anion exchange membrane electrolyzers using baseline materials and operation. *ACS Appl. Mater. Interfaces* **13**, 51917-51924 (2021).

96. Yang, C. et al. Mn-oxygen compounds coordinated ruthenium sites with deprotonated and low oxophilic microenvironments for membrane electrolyzer-based H_2-Production. *Adv. Mater.*, e2303331 (2023).

97. Zhao, Y. et al. Homologous NiCoP@NiFeP heterojunction array achieving high-current hydrogen evolution for alkaline anion exchange membrane electrolyzers. *J. Mater. Chem. A* **10**, 10209-10218 (2022).

98. Zhao, Y. et al. Operando reconstruction toward dual-cation-defects Co-containing NiFe oxyhydroxide for ultralow energy consumption industrial water splitting electrolyzer. *Adv. Energy Mater.* **13**, 2203595 (2023).
