# Supplementary material for: Flexible tungsten disulfide superstructure engineering for efficient alkaline hydrogen evolution in anion exchange membrane water electrolysers
Source: Nat Commun. 2024 Jul 8;15:5702. doi: 10.1038/s41467-024-50117-2 (PMC11231348; doi:10.1038/s41467-024-50117-2)
Supplement: Supplementary file 3 — Description of Additional Supplementary Files [file 41467_2024_50117_MOESM3_ESM.pdf]

Description Of Additional Supplementary File

Supplementary Dataset:

Supplementary Data 1. Atomic coordinates of the optimized computational models.

Supplementary Movies:

Supplementary Movie 1. Dynamic deformation of flexible WS2 superstructure in response to shear forces

Supplementary Movie 2. Molecular dynamic simulation of water molecules on stepped defect structures.

Supplementary Movie 3. In situ SEM mechanical properties test of WS2 superstructure (compression process).

Supplementary Movie 4. In situ SEM mechanical properties test of WS2 superstructure (stretching process).

Supplementary Movie 5. Wettability testing of different catalyst surfaces.

Supplementary Movie 6. Bubble release behavior on the electrode surfaces during HER electrocatalysis.

Supplementary Movie 7. H2 bubbles evolution on the asobtained electrode surface.
